# The Associations of *PMF1*, *ICAM1*, *AGT*, *TRIM65*, *FBF1*, and *ACOX1* Variants With Leukoaraiosis in Chinese Population

**DOI:** 10.3389/fgene.2019.00615

**Published:** 2019-07-23

**Authors:** Wen-Qing Huang, Hui-Ming Ye, Liang-Liang Cai, Qi-Lin Ma, Cong-Xia Lu, Sui-Jun Tong, Chi-Meng Tzeng, Qing Lin

**Affiliations:** ^1^Translational Medicine Research Center (TMRC), School of Pharmaceutical Sciences, Xiamen University, Xiamen, China; ^2^Shanghai Institute of Precision Medicine, Ninth People’s Hospital, Shanghai Jiao Tong University School of Medicine, Shanghai, China; ^3^Department of Clinical Laboratory, Women and Children’s Hospital, School of Medicine, Xiamen University, Xiamen, China; ^4^Department of Neurology and Center for Brain Research, The First Affiliated Hospital of Xiamen University, Xiamen, China; ^5^School of Medicine, Xiamen University, Xiamen, China; ^6^College of Pharmaceutical Sciences, Nanjing Tech University, Nanjing, China; ^7^Department of Neurology, The First Clinical Medical College and Graduate School of Fujian Medical University, Fuzhou, China

**Keywords:** association analysis, Chinese population, leukoaraiosis (LA), white matter lesions (WML), single nucleotide polymorphisms (SNPs)

## Abstract

**Background:** Leukoaraiosis (LA) is shown as white matter hyperintensities on T2-weighted magnetic resonance imaging brain scans. Together with candidate gene association studies (CGAS), multiple genome-wide association studies (GWAS) have reported large numbers of single nucleotide polymorphisms (SNPs) to be associated with LA in European populations. To date, no replication studies have been reported in independent Chinese samples.

**Methods:** Here, we performed a candidate gene association study comprising 220 Chinese subjects with LA and 50 controls. Thirty-nine polymorphisms on 32 risk genes were selected from previous studies, and they were genotyped through matrix-assisted laser desorption/ionization time-of-flight mass spectrometry (MALDI-TOF MS). Genetic association analysis was firstly performed in all subjects with LA. Then, the same analysis was conducted in the six random sampling cohorts of 50 LA patients, respectively. Data analyses on the associations of SNPs with LA risk were evaluated through Pearson’s χ^2^ and multivariate logistic regression tests.

**Results:** We found that eight polymorphisms in six genes (*PMF1*, *ICAM1*, *TRIM65*, *AGT*, *FBF1*, and *ACOX1*) were significantly associated with LA in the genetic association tests. Except for those eight gene variants, 24 other polymorphisms were not found to be significantly associated with LA in general genetic model, dominant model, recessive model, or multiplicative model. Among those eight polymorphisms, rs2984613 in *PMF1* showed significant association with LA in the cohort of 220 LA subjects, and such significant association remained in both general genetic model (OR: 0.262, 95% CI: 0.091–0.752, *p*_adj_ = 0.030) and recessive model (OR: 0.323, 95% CI: 0.119–0.881, *p*_adj_ = 0.038) when controlling for clinical variables. Seven other significant variants (rs5498 in *ICAM1*, rs699 in *AGT*, rs2305913 in *FBF1*, rs1135640 in *ACOX1*, and rs3760128, rs7214628, and rs7222757 in *TRIM65*) were identified in those six random sampling tests that were conducted in the adjusted cohorts of 50 LA patients. In addition, except for rs699 which showed detrimental effect and represented a risk variant for LA, seven other polymorphisms seemed to exert protective effects on LA and to reduce the risk of LA. It is necessary to confirm these associations in an independent cohort.

**Conclusions:** This first replication study on multiple genes in an independent Chinese population did not replicate any risk polymorphisms for LA other than rs 699 in *AGT* but revealed the significantly negative associations of *PMF1*, *ICAM1*, *TRIM65*, *FBF1*, and *ACOX1* polymorphisms with LA. It not only supported the strong ethnic differences in the genetics of LA but also indicated that those six identified genes may be involved in Chinese white matter lesions. Larger scales of CGAS and GWAS are necessary to confirm and decipher those ethnic-Han specific risk genes for LA in China.

## Introduction

Leukoaraiosis (LA) describes white matter hyperintensities (WMHs) or white matter lesions (WMLs) on FLAIR-MR brain scans in healthy elderly individuals (O’Sullivan, 2008; Xiong and Mok, 2011; Grueter and Schulz, 2012). It is commonly seen in elderly people with the prevalence from 50% to 100% depending on study population and study design (Liao et al., 1996; Longstreth et al., 1996; de Leeuw et al., 2001; Launer et al., 2006; Wen et al., 2009). LA does not remain asymptomatic when it progresses to a large portion of cerebral white matter region (Helenius and Tatlisumak, 2007; Kim et al., 2008; Debette and Markus, 2010). Clinically, LA was shown to be related with a variety of neuropsychiatric disorders and significantly increase the risk of stroke, depression, dementia, Parkinson’s disease (PD), multiple sclerosis (MS), or schizophrenia (Kim et al., 2008; Debette and Markus, 2010; Lin et al., 2015). To date, the pathogenesis underlying the well-known demyelination pathology of LA is poorly understood. However, a large account of susceptible genes (up to 50 genes) has been identified in white matter lesions, indicating the strong genetics of LA seen on MRI (Turner et al., 2004; Paternoster et al., 2009; Assareh et al., 2011; Fornage et al., 2011; Lin et al., 2015; Lopez et al., 2015). This is consistent with the high heritability reported in a previous male twins study and Framingham Heart Study (Carmelli et al., 1998; Atwood et al., 2004; Turner et al., 2004). Together with the results from the previous candidate gene association studies (CGAS) (Paternoster et al., 2009; Tran et al., 2016), the recent findings from two genome-wide association studies (GWAS) of WMLs supported the multifactorial nature of LA (Fornage et al., 2011; Verhaaren et al., 2015). Confusingly, those risk genes and single nucleotide polymorphisms (SNPs) (such as rs7412 and rs429358 in *ApoE*, rs699 in *AGT*, rs1799983 in *NOS3*, and rs662 and rs854560 in *PON1*) for LA identified in the CGAS could not be confirmed in both GWAS amongst European or multi-ethnic populations (Schmidt et al., 2000; Henskens et al., 2005; van Rijn et al., 2006; Fornage et al., 2007; Hogh et al., 2007; van Rijn et al., 2007; Taylor et al., 2008; Godin et al., 2009; Paternoster et al., 2009; Fernandez-Cadenas et al., 2011; Verhaaren et al., 2015). Although those two GWAS of LA consistently confirmed the risk chr17q25 locus for LA, the genome-wide significant risk factors (such as rs3744028 in *TRIM65*, rs1055129 in *TRIM47*, and rs11869977 in *WBP2*) identified in the first GWAS of LA in European descent could not be replicated by the multi-ethnic GWAS (Fornage et al., 2011; Verhaaren et al., 2015). On the contrary, the later GWAS in multi-ethnic individuals not only identified several genome-wide significant SNPs (such as rs3760128 and rs1551619 in *TRIM65*, and rs1135889 in *FBF1*) on chr17q25 locus, which only showed suggestive significance in the former GWAS, but also revealed four additional novel loci for LA on chr1q22 (rs2984613 in *PMF1*), chr2p16 (rs78857879 in *EFEMP1*), chr2p21 (rs11679640 in *HAAO*), and chr10q24 (rs12357919 and rs7909791 in *SH3PXD2A*) (Verhaaren et al., 2015).

In our published paper, we performed the first replication studies on the genetics of LA in the Han-Chinese individuals through pyrosequencing and restriction fragment length polymorphism (RFLP) (Huang et al., 2016). However, we failed to confirm the susceptibility of four risk SNPs (rs3744028 in *TRIM65*, rs1055129 in *TRIM47*, rs1135889 in *FBF1*, and rs1052053 in *PMF1*) identified in the first GWAS of LA. Those inconsistent findings may be due to the ethnic differences in the genetics of LA. To date, no replication studies have been conducted to confirm the data from the multi-ethnic GWAS and CGAS of LA in Asians.

As a developing country in Asia, China has a rapid development speed of economy and society during the past 30 years. People have great improvement in living condition and health care and prolonged life expectancies. With economic development and population aging, China with the largest population worldwide is increasingly bearing the challenge of the growing burden from neuropsychiatric disorders including stroke, depression, and dementia (such as Alzheimer’s disease) (Gu et al., 2013; Wang X. et al., 2017; Wang Q. et al., 2017; Wang W. et al., 2017). Given the increased risk of those diseases by LA and its unknown genetic architecture in the Chinese population, as well as its high incidence in Chinese hospitalized patients (Lin Q. et al., 2017), we attempt to decipher the genetic mechanism of LA and provide a cue for the prevention of LA and its associated neuropsychiatric diseases. In order to firstly confirm the ethnic differences and identify the Chinese descent-specific risk factors for LA, we re-selected 39 risk SNPs reported in previously published papers (**Table 1**) and performed a systematic association analysis of those SNPs with the risk of LA in one sample group from mainland China. Thus, the aim of the replication study is not only to confirm the ethnic differences in the genetics of LA but also to identify the susceptible SNPs in suspicious genes for LA amongst Chinese individuals.

**Table 1:** Information of selected 39 variants on 32 risk genes for leukoaraiosis (LA).

Gene	Locus	Gene description	SNP	Location	Function	Population	Source
*TRIM65*	17q25.1	Tripartite motif containing 65	rs7214628	Intron	—	European and Mix	*p* < 5E-8 in multi-ethnic GWAS (Verhaaren et al., 2015)
*NEURL*	10q24.33	Neuralized E3 ubiquitin protein ligase 1	rs72848980	Intron	—	European and Mix	
*PDCD11*	10q24.33	Programmed cell death 11	rs7894407	Intron	—	European and Mix	
*SH3PXD2A*	10q24.33	SH3 and PX domain-containing protein 2A	rs12357919	Intron	—	European and Mix	
			rs7909791	Intron	—	European and Mix	
*EFEMP1*	2p16.1	EGF containing fibulin-like extracellular matrix protein 1	rs78857879	Intron	—	Mix	
*PMF1*	1q22	Polyamine-modulated factor 1	rs2984613	Intron	—	Mix	
*UNC13D*	17q25.1	unc-13 homolog D (*C. elegans*)	rs1135688	Exon	K867E	European	*p <* 5E-8 in multi-ethnic GWAS (Verhaaren et al., 2015)
*TRIM47*		Tripartite motif containing 47	rs4600514	Exon	R187W	European	
*TRIM65*	17q25.1	Tripartite motif containing 65	rs3760128	Exon	L509P	European	
			rs7222757	Exon	V222G	European	
*FBF1*	17q25.1	Fas (TNFRSF6) binding factor 1	rs2305913	Exon	R151G	European	
*ACOX1*	17q25.1	Acyl-CoA oxidase 1	rs1135640	Exon	I312M	European	
*TAF5*	10q24.33	TATA-box binding protein associated factor 5	rs10883859	Exon	S130A	European	
*NBEAL1*	2q33.2	Neurobeachin like 1	rs72934505	Intron	—	European or Mix	*p* < 1E-5 in multi-ethnic GWAS and *p* < 5E-8 in GWAS in stroke patients (Verhaaren et al., 2015; Traylor et al., 2016)
*EVL*	17q25.1	Enah/Vasp-like	rs941898	Intron	—	European or Mix	
*C1QL1*	17q21.31	Complement component 1, q subcomponent-like 1	rs962888	Intergenic	—	European or Mix	
*COL4A2*	13q34	Collagen, type IV, alpha 2	rs9515201	Intron	—	European or Mix	
*APOE*	19q13.32	Apolipoprotein E	rs429358	Exon	C130R	Denmark/French/Pittsburgh	Adjusted *p* < 0.05 in independent CGAS studies (Schmidt et al., 2000; Henskens et al., 2005; van Rijn et al., 2006; Fornage et al., 2007; Hogh et al., 2007; van Rijn et al., 2007; Taylor et al., 2008; Godin et al., 2009; Paternoster et al., 2009; Fernandez-Cadenas et al., 2011; Lin et al., 2015)
			rs7412	Exon	R176C		
*ACE*	17q23.3	Angiotensin-converting enzyme	rs1799752	Intron	Ins/Del	UK (white)	
*AGT*	1q42.2	Angiotensinogen	rs699	Exon	M268T	Rotterdam	
*MMP3*	11q22.2	Matrix metalloproteinase 3	rs679620	Exon	K45E	Non-Hispanic Whites	
*MMP9*	20q13.12	Matrix metalloproteinase 9	rs2250889	Exon	R574P		
*ADD1*	11q22.2	Adducin 1	rs4961	Exon	G460W	Rotterdam	
*NOS3*	7q36.1	Endothelial nitric oxide synthase	rs1799983	Exon	D298E	Netherlands/Spain	
*ICAM1*	19p13.2	Intercellular adhesion molecule 1	rs5498	Exon	K469E		
*PON1*	7q21.3	Paraoxanase 1	rs854560	Exon	L55M	Australian/Spain/Greek	
			rs662	Exon	Q192R		
*A2M*	12p13.31	Alpha-2-Macroglobulin	rs669	Exon	I1000V	Spain (White)	
*BDNF*	11p14.1	Brain-derived neurotrophic factor	rs6265	Exon	V66M	United States	
*HFE*	6p22.2	Hemochromatosis	rs1799945	Exon	H63D	UK	
			rs1800562	Exon	C282Y		
*IL6*	7p15.3	Interleukin-6	rs1800795	5’UTR	—	USA (Whites)	Adjusted *p* < 0.05 in independent CGAS studies (Fornage et al., 2008; Fernandez-Cadenas et al., 2011; Shan et al., 2013; Yadav et al., 2014)
*IL5RA*	3p26.2	Interleukin 5A receptor	rs2290608	5’UTR	—	Spain (White)	
*PTGS2*	1q31.1	Cyclooxygenase-2 (COX2)	rs689466	TSS1500	—	Chinese	
			rs20417	TSS800	—		
*AQP4*	18q11.2	Aquaporin 4	rs2075575	Intron	—	Korea	
			rs9951307	Down -stream	—		

EGF, Epidermal Growth Factor.

## Methods

### Study Subjects

A total of 270 subjects which included 220 cases with LA and 50 controls were recruited from the department of neurology, the first affiliated hospital of Xiamen University, Fujian province, China, between April 2011 and September 2016 (**Table 2**). All participants were included in this study according to the **inclusion criteria and exclusion criteria as described in our **published article (Huang et al., 2016; Lin Q. et al., 2017)**.** The diagnoses of LA were independently made by the neurology specialists (QL, Q-LM, C-XL, and S-JT). Clinical data regarding gender, age, homocysteine (HCY), cholesterol, triglycerides (TG), low-density lipoprotein-cholesterol (LDL-C), high-sensitivity C-reactive protein (HCRP), hyperlipidemia, hypertension, and diabetes were collected and analyzed. The evaluation of risk factors (e.g., hypertension, diabetes, and hyperlipidemia) in both cases and controls was conducted according to the World Health Organization (WHO) guidelines for hypertension, diabetes and hyperlipidemia (Naito, 1975; Alberti and Zimmet, 1998; Guidelines Subcommittee, 1999). The study was approved by the ethics committee of Xiamen University. All study subjects provided written informed consent for genetic analysis and publication.

**Table 2:** Clinical characteristics of the study population.

	All (*n* = 270)	LA (*n* = 220)	Controls (*n* = 50)	OR (95% CI)	*P*-value
**Age (years)**	73.26 ± 10.68	74.64 ± 9.52	67.16 ± 13.19	1.068 (1.036, 1.100)	<0.001***^a^
**Male, *n* (%)**	157 (58.1%)	132 (60.0%)	25 (50.0%)	0.667 (0.360, 1.235)	>0.05
**Hypertension, *n* (%)**	188 (69.6%)	163 (74.1)	25 (50.0%)	2.860 (1.521, 5.375)	<0.001***
**Diabetes mellitus, *n* (%)**	65 (24.1%)	58 (26.4)	7 (14.0%)	2.199 (0.937, 5.162)	>0.05
**HCY (㩈µmol/L)**	16.49 ± 9.31	16.58 ± 8.79	16.09 ± 11.47	1.006 (0.970, 1.044)	>0.05 ^b^
**LDL-C (mmol/L)**	3.09 ± 1.02	3.01 ± 0.97	3.45 ± 1.16	0.661 (0.488, 0.897)	<0.01**^a^
**Cholesterol (mmol/L)**	4.89 ± 1.29	4.81 ± 1.26	5.22 ± 1.42	0.792 (0.623, 1.006)	>0.05^b^
**Triglyceride (mmol/L)**	1.33 ± 0.81	1.33 ± 0.81	1.34 ± 0.83	0.987 (0.667, 1.462)	>0.05^b^
**HCRP (mg/L) **	6.41 ± 5.26	6.39 ± 4.88	6.45 ± 6.81	0.998 (0.938, 1.062)	>0.05^b^

LA, leukoaraiosis; HCY, homocysteine; LDL-C, low-density lipoprotein cholesterol; HCRP, high-sensitivity C-reactive protein; OR, odds ratio; CI, conﬁdence intervals.

*Signiﬁcant association with two-sided p value < 0.05, **p < 0.01, ***p < 0.001. a and b indicate that the p-value estimate from binary logistic regression analysis could be confirmed by t test and nonparametric Wilcoxon test, respectively.

### Gene Selection

In genetic studies, those variants that could directly contribute to the pathology by affecting the structure and function of protein or that may act as biomarkers for human diseases due to strongly significant associations may be more interesting than those polymorphisms located in the non-coding regions of genes (such as promoter, 5′UTR, 3′UTR) or intergenic regions, although the latter could modify gene expression through regulating transcriptional activity or messenger RNA (mRNA) stability and further contribute to diseases. We thus selected 39 interesting SNPs to perform a systematic association analysis on the genetics of LA according to the potential significance and function of polymorphisms. Those candidate SNPs were composed of three groups. The first group included 18 SNPs (**Table 1**), and they were chosen from 45 risk SNPs which consisted of 30 polymorphisms with the GWAS significance of *p* < 5.0 × 10^–8^ and 15 polymorphisms showing suggestive association with LA (*p* < 5.0 × 10^–8^ < *p* < 1.0 × 10^–5^) (**Supplemental Table 1**) (Verhaaren et al., 2015). Twenty-seven of 45 polymorphisms were excluded in the present study due to weak association with LA or unclear function in gene regulation. Among those selected genetic polymorphisms, seven SNPs occurring in gene bodies were selected from those eight polymorphisms which showed genome-wide significance (*p* < 5.0 × 10^–8^) in a multi-ethnic (Mix) population. They included rs7214628 in *TRIM65*, rs72848980 in *NEURL*, rs7894407 in *PDCD11*, rs12357919 in *SH3PXD2A*, rs7909791 in *SH3PXD2A*, rs78857879 in *EFEMP1*, and rs2984613 in *PMF1*. Seven additional missense SNPs located in the protein-encoding regions of genes were selected from 22 polymorphisms that exhibited genome-wide significance (*p* < 5.0 × 10^–8^) only in a European population. Those putatively functional SNPs (rs1135688 in *UNC13D*, rs4600514 in *TRIM47*, rs3760128 and rs7222757 in *TRIM65*, rs2305913 in *FBF1*, rs1135640 in *ACOX1*, and rs10883859 in *TAF5*) could disrupted the structure and function of the protein. Four additional SNPs (rs72934505 in *NBEAL1*, rs941898 in *EVL*, rs962888 in *C1QL1*, and rs9515201 in *COL4A2*) were selected from 15 polymorphisms with highly suggestive significance (*p* < 5.0 × 10^–8^ < *p* < 1.0 × 10^–5^) in multi-ethnic GWAS because they showed GWAS significance of *p* < 5.0 × 10^–8^ in another genome-wide meta-analysis of white matter hyperintensity volumes (WMHV) in stroke patients (Verhaaren et al., 2015; Traylor et al., 2016). In addition, 15 SNPs were included in the second group (**Table 1**). Since they resulted in the missense changes, those SNPs were chosen from 28 SNPs which were reported to be related with the risk of WMHs or WMLs in previous candidate gene association studies amongst European or American individuals and listed in **Supplemental Table 1** (Schmidt et al., 2000; Henskens et al., 2005; van Rijn et al., 2006; Fornage et al., 2007; Hogh et al., 2007; van Rijn et al., 2007; Taylor et al., 2008; Godin et al., 2009; Paternoster et al., 2009; Fernandez-Cadenas et al., 2011; Shan et al., 2013; Lin et al., 2015). Eleven of 28 SNPs were excluded from the present study since they were located in the non-coding regions of genes and considered to be non-functional SNPs. Two additional SNPs were discarded because of the technical difficulties in detection. Given that more and more evidence supported LA as an inflammation-associated disease in the central nervous system (CNS) (Wright et al., 2009; Lin et al., 2015; Lin H. et al., 2017; Huang et al., 2018), we thought that those polymorphisms which occurred in immune/inflammation-related genes may contribute to the susceptibility of LA. Thus, six recently reported risk SNPs of immune/inflammation-related genes which showed significant association with LA were included in the last group (Fornage et al., 2008; Fernandez-Cadenas et al., 2011; Shan et al., 2013; Yadav et al., 2014). Those polymorphisms occur on the introns of genes. The gene list is presented in **Table 1** and **Supplemental Table 1**.

### Genetic Analysis

**Figure 1:**
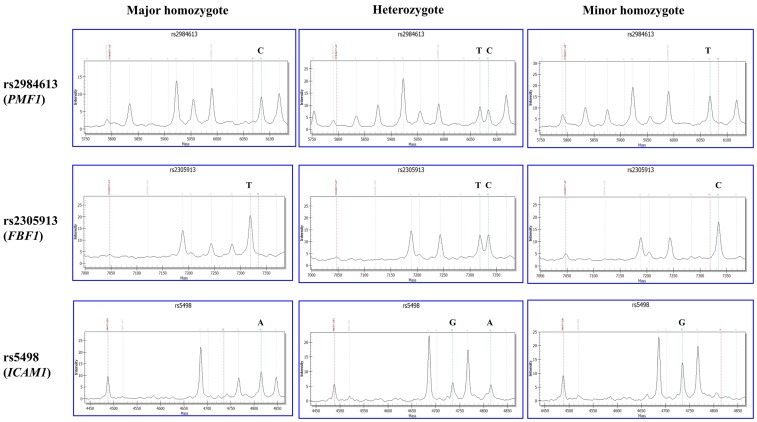
Matrix-assisted laser desorption/ionization time-of-flight mass spectrometry (MALDI-TOF MS)-based genotyping of rs2984613 in *PMF1*, rs2305913 in *FBF1*, and rs5498 in *ICAM1*. The *X* and *Y* axes represent the mass and intensity of extension product, respectively. The red dotted line on the left indicates the mass of extension primer. The bean green dotted line on the right indicates the extension product on the major or minor allele. The left and right columns show the MS result of major homozygote (wild type) and minor homozygote (mutant), respectively. The middle column shows the MS result of heterozygote.

Genomic DNA was extracted from whole blood of the subjects using the MagCore Genomic DNA Whole Blood Kit (RBC Bioscience, Taiwan, China). The quality and quantity were measured by NanoDrop2000 spectrophotometer. All extracted DNA samples were stored in aliquots at −20°C for further requirement. Genotyping for all candidate SNPs was performed by polymerase chain reaction (PCR) and matrix-assisted laser desorption/ionization time-of-flight mass spectrometry (MALDI-TOF MS) using the MassARRAY^®^ MALDI-TOF System (Sequenom Inc., San Diego, CA). Firstly, polymerase chain reaction (PCR) was used to amplify the targeted sequence of SNP from each DNA sample through locus-specific primers which were designed using the Mass ARRAY Assay Design 3.0 software. Secondly, PCR products were extended by SNP-specific extension primers. Thirdly, final base extension products were further treated using clean resin (Sequenom). Fourthly, final purified products were transferred onto the 384 format microarray (MassARRAY SpectroCHIP, Sequenom), then the genotype of each SNP-specific extension product was detected using MassARRAY Analyzer Compac. Those 39 SNPs were divided into two groups for genotyping. One group consisted of 24 SNPs (rs1135640, rs1135688, rs12357919, rs1799945, rs2075575, rs2290608, rs2305913, rs2984613, rs3760128, rs4961, rs4600514, rs7412, rs5498, rs662, rs669, rs679620, rs689466, rs699, rs7214628, rs78857879, rs854560, rs9515201, rs72934505, and rs962888). The other group included 15 SNPs (rs1799983, rs1800795, rs20417, rs2250889, rs429358, rs6265, rs7222757, rs72848980, rs7894407, rs7909791, rs941898, rs9951307, rs1799752, rs1800562, and rs10883859). Finally, the genotyping data we collected were further analyzed by MassARRAY TYPER software (Sequenom Inc., San Diego, CA). The MS-based genotyping results of three selected SNPs (rs2984613 in *PMF1*, rs2305913 in *FBF1*, and rs5498 in *ICAM1*) are shown in **Figure 1**. Genotyping data of other SNPs are shown in the **Supplemental Figure 1**. All primers were designed by MassARRAY Assay Designer 3.1 (Sequenom, Inc., San Diego, CA) and produced by Sangon Biotech^®^.

### Statistical Analysis

Clinical and biochemical data were expressed as mean ± SD or percentage. The Pearson’s chi-square (χ^2^) test was used to compare the difference in qualitative variables including sex, hypertension, diabetes, and hyperlipidemia between subjects with LA and controls without LA. Difference in those quantitative variables including age, cholesterol, triglycerides, homocysteine (HCY), low-density lipoprotein-cholesterol (LDL-C), and high-sensitivity C-reactive protein (HCRP) between LA group and control group was assessed through binary logistic regression analysis. Furthermore, nonparametric Wilcoxon test was conducted to compare the difference in those variables (including cholesterol, triglycerides, HCY, and HCRP) that could not pass through the test of normality between cases and controls. *t* test was used to confirm the effect of the clinical variables (such as age and LDL-C level) on LA. Every SNP was assessed for deviation from the Hardy–Weinberg equilibrium in cases and controls using an exact test as implemented in the Finetti program (http://ihg.gsf.de/cgi-bin/hw/hwa1.pl) before association analysis for all SNPs. Four genetic models, including the general genetic model, the dominant model, the recessive model, and the multiplicative model (allele test) were applied to analyze the influence of every SNP on LA. We used Pearson’s chi-square (㩈χ^2^) and Fisher’s tests to test the differences in frequencies of the alleles and genotypes between cases and controls. The multivariate adjustments of the associations were performed in the binary logistic regression model. We adjusted analyses for age, hypertension, and LDL-C level by including those significant variables associated with LA together with single SNP into covariates in binary logistic regression model and using “Enter” method to perform covariates screening. Wald test was used in the hypothesis test of regression coefficient. Odds ratio (OR) and 95% confidence interval (95% CI) were applied for the evaluation. Power was calculated by the OpenEpi tool (http://www.openepi.com). All statistical analyses were performed using SPSS 17.0 software (SPSS Inc., Chicago, IL, USA), and a two-tailed *p* value <0.05 was considered to be statistically significant.

## Results

### Characteristics of Study Population

This study population consisted of 220 subjects with LA and 50 subjects without LA. All the subjects were ethnic Han-Chinese. The clinical characteristics of those subjects are summarized in **Table 2**. The mean age was 73.26 ± 10.68 years, and 58.1% of the subjects were males. The percentages of subjects with history of hypertension and diabetes mellitus were 69.6% and 24.1%, respectively. There were not significant differences in gender (*p* > 0.05), the history of diabetes mellitus (*p* > 0.05), smoking (*p* > 0.05) and the levels of homocysteine (HCY, *p* > 0.05), cholesterol (*p* > 0.05) and triglyceride (*p* > 0.05), as well as the level of high-sensitivity C-reactive protein (HCRP, p > 0.05), but in age (*p* < 0.001), the history of hypertension (p < 0001) and the level of low-density lipoprotein cholesterol (LDL-C, *p* < 0.01) between LA group and control group (**Supplemental Figure 2**). Among those significant clinical variables, both age and the percentage of hypertension in subjects with LA were significantly higher than those in the controls, while controls showed significantly higher levels of LDL-C than LA subjects. Consistent with the findings in our previous study (Lin Q. et al., 2017), these results not only confirmed age (OR 1.068, 95% CI 1.036–1.100, *p* < 0.001) and hypertension (OR 2.860, 95% CI 1.521–5.375, *p* < 0.001) as the risk factors for LA, but also suggested high level of LDL-C (OR 0.661, 95% CI 0.488–0.897, *p* < 0.01) as protective factor for LA.

### Association Analysis of Alleles and Genotypes With Risk of LA

We used MALDI-TOF MS technology to detect 39 selected SNPs in 270 samples. Among those SNPs, seven polymorphisms (rs72934505 in *NBEAL1*, rs10883859 in *TAF5*, rs4600514 in *TRIM47*, rs7909791 in *SH3PXD2A*, rs1800562 in *HFE*, rs1799752 in *ACE*, and rs7412 in *APOE*) failed in the genotyping. Finally, 32 SNPs were genotyped successfully in the present study. All genotyping data are summarized in **Supplemental Table 2**. In order to determine whether the genotype frequencies of two alleles were consistent in patients and controls, we firstly used Chi-square test to assess the Hardy–Weinberg equilibrium (HWE). Except those four SNPs that showed very low frequency in Asians in 1000 Genomes database and hardly occurred in the present cohort (rs72848980 in *NEURL*, rs12357919 in *SH3PXD2A*, rs78857879 in *EFEMP1*, and rs1800795 in *IL6*), all SNPs met the HWE criterion in both groups (*p* > 0.05).

According to the allele distributions between LA and controls, we further used the multiplicative model to examine the association of every SNP with the risk of LA. Unfortunately, all of candidate SNPs did not show significant association with LA when Bonferroni-corrected alpha level of 0.00139 was considered (**Table 3**).

**Table 3:** Statistical analysis of allele frequencies among LA subjects and controls.

SNPs	Gene	Minor allele	LA (220)		Control (50)		OR (95% CI)	*P* value	*P_c_*
			No.	%	No.	%			
**rs7214628 (A > G)**	**TRIM65**	G	61	13.9	15	15.0	0.912 (0.495, 1.682)	0.768	1.000
**rs72848980 (G > A)**	**NEURL**	A	0	0	0	0	—	—	—
**rs7894407 (T > C)**	**PDCD11**	C	180	40.9	40	40.0	1.038 (0.667, 1.617)	0.867	1.000
**rs12357919 (T > C)**	**SH3PXD2A**	C	0	0	0	0	—	—	—
**rs78857879 (G > A)**	**EFEMP1**	A	2	0.5	0	0	—	—	—
**rs2984613 (C > T)**	**PMF1**	T	105	23.9	36	36.0	0.557 (0.351, 0.886)	0.013*	0.416
**rs1135688 (T > C)**	**UNC13D**	C	205	46.6	50	50.0	0.872 (0.565, 1.347)	0.538	1.000
**rs3760128 (A > G)**	**TRIM65**	G	104	23.6	32	32.0	0.658 (0.409, 1.057)	0.082	1.000
**rs7222757 (A > C)**	**TRIM65**	C	103	23.4	30	30.0	0.713 (0.441, 1.154)	0.167	1.000
**rs2305913 (T > C)**	**FBF1**	C	95	21.5	31	31.0	0.613 (0.379, 0.991)	0.045*	1.000
**rs1135640 (C > G)**	**ACOX1**	G	83	18.9	27	27.0	0.629 (0.381, 1.038)	0.068	1.000
**rs941898 (T > G)**	**EVL**	G	76	17.3	19	19.0	0.890 (0.510, 1.554)	0.682	1.000
**rs962888 (G > A)**	**C1QL1**	A	134	30.5	38	38.0	0.714 (0.455, 1.123)	0.144	1.000
**rs9515201 (C > A)**	**COL4A2**	A	48	11.0	8	8.0	1.415 (0.647, 3.094)	0.382	1.000
**rs429358 (T > C)**	**APOE**	C	38	8.6	13	13.0	0.633 (0.323, 1.238)	0.178	1.000
**rs699 (G > A)**	AGT	A	82	18.6	16	16.0	1.203 (0.669, 2.161)	0.537	1.000
**rs679620 (C > T)**	**MMP3**	T	133	30.5	31	31.0	0.977 (0.610, 1.564)	0.923	1.000
**rs2250889 (C > G)**	**MMP9**	G	103	23.4	22	22.0	1.084 (0.643, 1.826)	0.763	1.000
**rs4961 (G > T)**	**ADD1**	T	188	42.7	47	47.0	0.841 (0.544, 1.301)	0.437	1.000
**rs1799983 (G > T)**	**NOS3**	T	49	11.1	10	10.0	1.128 (0.550, 2.312)	0.742	1.000
**rs5498 (A > G)**	**ICAM1**	G	111	25.3	38	38.0	0.554 (0.350, 0.875)	0.011*	0.352
**rs854560 (A > T)**	**PON1**	T	12	2.7	3	3.0	0.907 (0.251, 3.274)	1.000	1.000
**rs662 (C > T)**	**PON1**	T	165	37.5	37	37.0	1.022 (0.652, 1.601)	0.926	1.000
**rs669 (T > C)**	**A2M**	C	46	10.5	15	15.0	0.662 (0.353, 1.240)	0.195	1.000
**rs6265 (C > T)**	**BDNF**	T	213	48.4	49	49.0	0.977 (0.633, 1.508)	0.915	1.000
**rs1799945 (C > G)**	**HEE**	G	20	4.5	3	3.0	1.540 (0.449, 5.286)	0.677	1.000
**rs1800795 (G > C)**	**IL6**	C	0	0	0	0	—	—	—
**rs2290608 (C > T)**	**IL5RA**	T	85	19.3	21	21.0	0.901 (0.527, 1.540)	0.702	1.000
**rs689466 (T > C)**	**COX2**	C	217	49.5	44	44.0	1.250 (0.807, 1.935)	0.317	1.000
**rs20417 (C > G)**	**COX2**	G	19	4.3	4	4.0	1.083 (0.360, 3.256)	1.000	1.000
**rs2075575 (G > A)**	**AQP4**	A	102	23.2	27	27.0	0.816 (0.498, 1.337)	0.419	1.000
**rs9951307 (A > G)**	**AQP4**	G	73	16.7	15	15.0	1.133 (0.620, 2.073)	0.684	1.000

OR, odds ratios; CI, conﬁdence interval; D, dominant model; R, recessive model; Pc, adjusted p value after Bonferroni correction for multiple tests. *Signiﬁcant association with two-tailed p value < 0.05.

Given the significant associations of the alleles of rs2984613 in *PMF1* (OR 0.557, 95% CI 0.351–0.886, *p* = 0.013), rs2305913 in *FBF1* (OR 0.613, 95% CI 0.379–0.991, *p* = 0.045) and rs5498 in *ICAM1* (OR 0.554, 95% CI 0.350–0.875, *p* = 0.011) with LA before multiple corrections, we performed the univariate and multivariate analyses to assess the association of those three SNPs with the risk of LA in the general genetic model, the dominant model, and the recessive model. As shown in **Table 4**, LA was not observed to be significantly associated with rs2305913 (*p* > 0.05), but it was shown to be significantly associated with rs2984613 (OR 0.323, 95% CI 0.119–0.881, *p* = 0.047) and rs5498 (OR 0.478, 95% CI 0.255–0.898, *p* = 0.020) in the recessive model and the dominant model, respectively. Moreover, rs2984613 still showed significant association (*p*_adj_ = 0.030 for minor homozygote, *p*_adj_ = 0.038 for major allele carriers) with LA after controlling for those significant variables including age, hypertension, and LDL-C. The power for rs2984613 was up to 60.32% in the present study. This indicated the potential effect of rs2984613 on reducing LA risk.

**Table 4:** Associations between three candidate gene polymorphisms and the risk of LA.

Gene	SNPs	Location/amino acid change	Genotype	LA (*n* = 220)		Control (*n *= 50)		OR (95% CI)	P	*P*adj	Power (%)
				No.	%	No.	%				
**PMF1**	rs2984613 (C > T)	Intron	CC	126	57.3	21	42.0	**1**			
			CT	83	37.7	22	44.0	0.629 (0.325, 1.215)	0.165	0.506	13.12
			TT	11	5.0	7	14.0	0.262 (0.091, 0.752)	0.022*	0.030*	60.32
			CT+TT (D)	94	42.7	29	58.0	0.540 (0.290, 1.006)	0.050	0.218	50.05
			CC+CT (R)	209	95.0	43	86.0	0.323 (0.119, 0.881)	0.047*	0.038*	60.32
**FBF1**	rs2305913 (T > C)	Exon/R151G	TT	134	60.9	24	48.0	**1**			
			TC	77	35.0	21	42.0	0.657 (0.343, 1.257)	0.202	0.169	15.60
			CC	9	4.1	5	10.0	0.322 (0.099, 1.045)	0.111	0.150	41.87
			TC+CC (D)	86	39.1	26	52.0	0.592 (0.320, 1.098)	0.094	0.100	38.75
			TT+TC (R)	211	95.9	45	90.0	0.384 (0.123, 1.200)	0.178	0.250	41.87
**ICAM1**	rs5498 (A > G)	Exon/K469E	AA	123	56.2	19	38.0	**1**			
			AG	81	37.0	24	48.0	0.521 (0.268, 1.013)	0.052	0.181	30.43
			GG	15	6.8	7	14.0	0.331 (0.119, 0.917)	0.059	0.092	40.87
			AG+GG (D)	96	43.8	31	62.0	0.478 (0.255, 0.898)	0.020*	0.093	64.62
			AA+AG (R)	204	93.2	43	86.0	0.452 (0.174, 1.174)	0.168	0.177	40.87

All P values were calculated in relation to the major homozygote genotype. OR, odds ratios; CI, conﬁdence interval; D, dominant model; R, recessive model; P_adj_, adjusted p value which was corrected for clinical variables (age, hypertension, and LDL-C levels). *Signiﬁcant association with two-tailed p value < 0.05.

Due to the quadruple size difference between the control cohort and the cohort of LA subjects, the above regular association analysis could lead to some false positives and false negatives. In order to validate rs2984613 in *PMF1* and rs5498 in *ICAM1* and identify more significantly associated variants, we adjusted the cohort size of LA subjects to the size of the control cohort by decreasing the cohort size of LA subjects from 200 to 50, then performed a comprehensive association analyses on all candidate SNPs. In order to increase the reliability of results, we conducted six random sampling tests by randomly selecting 50 cases from 220 subjects with LA. The clinical characteristics of those randomly selected subjects in the sampling tests are summarized in **Supplemental Table 3**. As shown in **Table 5**, both rs2984613 in *PMF1* and rs5498 in *ICAM1* showed significant associations with LA in up to four random sampling tests. Additionally, six polymorphisms (rs699 in *AGT*, rs2305913 in *FBF1*, rs1135640 in *ACOX1*, and rs3760128, rs7214628, and rs7222757 in *TRIM65*) were observed to be significantly associated with LA. Moreover, four of six SNPs (rs699, rs2305913, rs1135640, and rs3760128) showed significant associations in more than or equal to two random sampling tests. Among those eight polymorphisms identified in the random sampling tests, only five variants (rs5498 in *ICAM1*, rs699 in *AGT*, rs2305913 in *FBF1*, rs1135640 in *ACOX1*, and rs3760128 in *TRIM65*) still showed significant associations with LA after adjusting for those associated variables in up to two random sampling tests. Furthermore, we surprisingly found that only rs699 showed significantly positive association with LA (OR 2.602, 95% CI 1.096–6.175, padj = 0.030 in the first sampling test; OR 2.904, 95% CI 1.102–7.656, padj = 0.031 in the fifth sampling test), suggesting this variant as the risk factor for LA. Therefore, those results indicated that rs699 in AGT and seven other SNPs (rs2984613 in *PMF1*, rs5498 in *ICAM1*, rs2305913 in *FBF1*, rs1135640 in *ACOX1*, and rs3760128, rs7214628 and rs7222757 in *TRIM65*) exert potentially detrimental and protective effect on LA, respectively.

**Table 5:** The association analysis of 32 candidate SNPs with LA in 6 random sampling tests.

SNPs	Gene	1st			2nd			3rd		
		OR (95% CI)	*P* value	*P*_adj_	OR (95% CI)	*P* value	*P*_adj_	OR (95% CI)	*P* value	*P*_adj_
**rs7214628 **	**TRIM65**	0.907 (0.382, 2.154)	0.826	0.999	0.820 (0.342, 1.966)	0.656	0.993	1.312 (0.569, 3.029)	0.524	0.278
**rs72848980**	**NEURL**	—	—	—	—	—	—	—	—	—
**rs7894407 **	**PDCD11**	1.043 (0.590, 1.844)	0.885	0.870	0.807 (0.453, 1.436)	0.465	0.534	1.043 (0.590, 1.844)	0.885	0.982
**rs12357919**	**SH3PXD2A**	—	—	—	—	—	—	—	—	—
**rs78857879**	**EFEMP1**	—	—	—	—	—	—	—	—	—
**rs2984613 **	**PMF1**	0.436 (0.224, 0.848)	0.014*	0.081	0.454 (0.237, 0.867)	0.017*	0.085	0.460 (0.238, 0.890)	0.021*	0.201
**rs1135688 **	**UNC13D**	1.131 (0.645, 1.983)	0.668	0.602	0.966 (0.574, 1.623)	0.895	0.952	0.811 (0.483, 1.364)	0.430	0.744
**rs3760128**	**TRIM65**	0.730 (0.385, 1.384)	0.334	0.482	0.621 (0.335, 1.151)	0.130	0.265	0.657 (0.345, 1.251)	0.201	0.377
**rs7222757**	**TRIM65**	0.771 (0.409, 1.455)	0.423	0.409	0.686 (0.372, 1.262)	0.225	0.388	0.732 (0.388, 1.384)	0.338	0.508
**rs2305913**	**FBF1**	0.731 (0.386, 1.384)	0.336	0.448	0.607 (0.322, 1.143)	0.122	0.224	0.644 (0.333, 1.245)	0.191	0.319
**rs1135640**	**ACOX1**	0.681 (0.353, 1.314)	0.252	0.308	0.622 (0.325, 1.192)	0.153	0.229	0.707 (0.361, 1.385)	0.312	0.426
**rs941898 **	**EVL**	1.000 (0.518, 1.930)	1.000	0.855	0.822 (0.404, 1.674)	0.589	0.855	0.592 (0.272, 1.288)	0.186	0.219
**rs962888**	**C1QL1**	0.907 (0.493, 1.672)	0.756	0.454	0.651 (0.340, 1.248)	0.196	0.493	0.771 (0.408, 1.456)	0.422	0.851
**rs9515201**	**COL4A2**	1.000 (0.391, 2.557)	1.000	0.834	1.449 (0.613, 3.423)	0.398	0.482	1.503 (0.610, 3.702)	0.375	0.538
**rs429358 **	**APOE**	0.426 (0.153, 1.186)	0.102	0.056	0.426 (0.153, 1.186)	0.102	0.132	0.577 (0.225, 1.478)	0.252	0.159
**rs699**	**AGT**	1.666 (0.815, 3.406)	0.162	0.030*	1.617 (0.808, 3.236)	0.174	0.066	1.233 (0.591, 2.571)	0.576	0.265
**rs679620**	**MMP3**	0.789 (0.431, 1.447)	0.444	0.340	0.917 (0.514, 1.635)	0.768	0.806	0.887 (0.510, 1.545)	0.672	0.745
**rs2250889**	**MMP9**	1.063 (0.536, 2.109)	0.861	0.477	1.258 (0.646, 2.452)	0.500	0.848	0.795 (0.369, 1.714)	0.558	0.390
**rs4961 **	**ADD1**	0.704 (0.392, 1.266)	0.241	0.438	0.916 (0.511, 1.639)	0.767	0.923	0.795 (0.438, 1.443)	0.451	0.638
**rs1799983**	**NOS3**	1.209 (0.513, 2.848)	0.664	0.428	1.114 (0.448, 2.768)	0.817	0.853	0.902 (0.370, 2.200)	0.820	0.640
**rs5498**	**ICAM1**	0.425 (0.220, 0.822)	0.011*	0.031*	0.490 (0.261, 0.919)	0.026*	0.098	0.522 (0.275, 0.990)	0.047*	0.123
**rs854560**	**PON1**	1.362 (0.289, 6.426)	0.696	0.527	0.653 (0.104, 4.085)	0.648	0.906	1.000 (0.192, 5.210)	1.000	0.767
**rs662 **	**PON1**	1.512 (0.828, 2.763)	0.178	0.580	1.227 (0.654, 2.304)	0.523	0.995	1.353 (0.723, 2.531)	0.345	0.823
**rs669**	**A2M**	0.447 (0.176, 1.138)	0.091	0.233	0.928 (0.433, 1.985)	0.846	0.656	0.758 (0.325, 1.768)	0.521	0.953
**rs6265**	**BDNF**	0.722 (0.424, 1.229)	0.230	0.310	0.931 (0.551, 1.573)	0.789	0.911	1.125 (0.649, 1.950)	0.675	0.885
**rs1799945**	**HEE**	1.362 (0.289, 6.426)	0.696	0.785	1.000 (0.192, 5.210)	1.000	0.441	1.362 (0.289, 6.426)	0.696	0.596
**rs1800795**	**IL6**	—	—	—	—	—	—	—	—	—
**rs2290608**	**IL5RA**	1.414 (0.722, 2.769)	0.312	0.244	1.000 (0.502, 1.992)	1.000	0.748	1.189 (0.610, 2.318)	0.611	0.310
**rs689466**	**COX2**	1.403 (0.788, 2.497)	0.250	0.318	1.303 (0.726, 2.339)	0.376	0.137	1.185 (0.669, 2.098)	0.562	0.236
**rs20417**	**COX2**	1.278 (0.322, 5.066)	0.727	0.731	1.000 (0.236, 4.241)	1.000	0.717	0.479 (0.084, 2.743)	0.409	0.366
**rs2075575**	**AQP4**	0.850 (0.445, 1.623)	0.622	0.718	0.750 (0.384, 1.464)	0.399	0.227	0.857 (0.457, 1.609)	0.631	0.251
**rs9951307**	**AQP4**	0.867 (0.413, 1.820)	0.707	0.496	1.184 (0.529, 2.650)	0.682	0.958	0.915 (0.401, 2.089)	0.833	0.604
SNPs	Gene	4th			5th			6th		
		OR (95% CI)	*P* value	*P*_adj_	OR (95% CI)	*P* value	*P*_adj_	OR (95% CI)	*P* value	*P*_adj_
**rs7214628**	**TRIM65**	0.380 (0.139, 1.035)	0.058	0.037*	0.907 (0.382, 2.154)	0.826	0.937	0.820 (0.342, 1.966)	0.656	0.432
**rs72848980**	**NEURL**	—	—	—	—	—	—	—	—	—
**rs7894407**	**PDCD11**	1.184 (0.669, 2.096)	0.562	0.304	1.464 (0.822, 2.608)	0.196	0.207	1.041 (0.596, 1.819)	0.887	0.628
**rs12357919**	**SH3PXD2A**	—	—	—	—	—	—	—	—	—
**rs78857879**	**EFEMP1**	—	—	—	—	—	—	—	—	—
**rs2984613**	**PMF1**	0.512 (0.267, 0.981)	0.044*	0.142	0.545 (0.294, 1.011)	0.054	0.127	0.545 (0.294, 1.011)	0.054	0.066
**rs1135688**	**UNC13D**	0.896 (0.528, 1.523)	0.686	0.686	0.700 (0.388, 1.265)	0.237	0.252	0.757 (0.433, 1.320)	0.326	0.442
**rs3760128**	**TRIM65**	0.546 (0.287, 1.038)	0.065	0.042*	0.511 (0.259, 1.006)	0.052	0.100	0.511 (0.259, 1.006)	0.052	0.037*
**rs7222757**	**TRIM65**	0.576 (0.304, 1.090)	0.090	0.045*	0.575 (0.295, 1.121)	0.104	0.171	0.575 (0.295, 1.121)	0.104	0.064
**rs2305913**	**FBF1**	0.480 (0.242, 0.954)	0.036*	0.016*	0.491 (0.245, 0.988)	0.046*	0.084	0.461 (0.228, 0.932)	0.031*	0.019*
**rs1135640**	**ACOX1**	0.436 (0.212, 0.895)	0.024*	0.019*	0.627 (0.317, 1.239)	0.179	0.216	0.448 (0.217, 0.960)	0.030*	0.032*
**rs941898**	**EVL**	0.938 (0.465, 1.891)	0.858	0.896	0.811 (0.389, 1.691)	0.576	0.778	1.318 (0.684, 2.539)	0.410	0.683
**rs962888**	**C1QL1**	0.685 (0.357, 1.314)	0.255	0.548	0.698 (0.371,1.316)	0.267	0.228	0.907 (0.493, 1.672)	0.756	0.869
**rs9515201**	**COL4A2**	1.855 (0.784, 4.385)	0.160	0.320	1.328 (0.561, 3.147)	0.519	0.495	1.711 (0.725, 4.040)	0.221	0.572
**rs429358**	**APOE**	0.928 (0.434, 1.982)	0.847	0.709	0.463 (0.174, 1.233)	0.123	0.113	0.577 (0.225, 1.478)	0.252	0.073
**rs699**	**AGT**	1.891 (0.920, 3.887)	0.083	0.146	1.411 (0.677, 2.939)	0.358	0.031*	1.447 (0.677, 3.092)	0.341	0.123
**rs679620**	**MMP3**	1.041 (0.596, 1.820)	0.887	0.673	0.955 (0.528, 1.728)	0.880	0.773	0.913 (0.505, 1.650)	0.763	0.913
**rs2250889**	**MMP9**	1.000 (0.493, 2.030)	1.000	0.296	1.000 (0.493, 2.030)	1.000	0.438	1.357 (0.681,2.702)	0.385	0.935
**rs4961**	**ADD1**	0.887 (0.510, 1.544)	0.672	0.553	0.693 (0.380, 1.263)	0.231	0.254	0.626 (0.340, 1.153)	0.133	0.078
**rs1799983**	**NOS3**	1.000 (0.416, 2.403)	1.000	0.977	1.093 (0.478, 2.496)	0.833	0.785	0.395 (0.121, 1.289)	0.124	0.285
**rs5498**	**ICAM1**	0.564 (0.303, 1.050)	0.071	0.273	0.404 (0.208, 0.783)	0.007**	0.028*	0.691 (0.378, 1.262)	0.229	0.584
**rs854560**	**PON1**	1.000 (0.192, 5.210)	1.000	0.825	0.320 (0.032, 3.184)	0.331	0.764	1.000 (0.192, 5.210)	1.000	0.601
**rs662 **	**PON1**	1.556 (0.803, 3.014)	0.190	0.522	0.703 (0.375, 1.317)	0.271	0.259	1.056 (0.553, 2.016)	0.869	0.691
**rs669**	**A2M**	0.684 (0.289, 1.620)	0.388	0.606	0.775 (0.344, 1.746)	0.539	0.891	0.613 (0.254, 1.479)	0.276	0.845
**rs6265**	**BDNF**	1.071 (0.640, 1.793)	0.793	0.931	1.262 (0.729, 2.183)	0.406	0.393	1.038 (0.606, 1.780)	0.891	0.573
**rs1799945**	**HEE**	1.741 (0.393, 7.713)	0.465	0.716	3.439 (0.872, 13.563)	0.078	0.260	1.741 (0.393, 7.713)	0.465	0.910
**rs1800795**	**IL6**	—	—	—	—	—	—	—	—	—
**rs2290608**	**IL5RA**	0.875 (0.427, 1.792)	0.715	0.925	1.000 (0.479, 2.088)	1.000	0.945	1.123 (0.576, 2.187)	0.734	0.203
**rs689466**	**COX2**	1.146 (0.634, 2.069)	0.652	0.106	1.241 (0.696, 2.212)	0.464	0.683	1.253 (0.694, 2.263)	0.455	0.053
**rs20417**	**COX2**	0.734 (0.156, 3.462)	0.696	0.487	1.872 (0.512, 6.848)	0.343	0.466	1.568 (0.414, 5.935)	0.508	0.996
**rs2075575**	**AQP4**	0.902 (0.481, 1.692)	0.749	0.456	0.814 (0.433, 1.530)	0.523	0.457	0.864 (0.467, 1.596)	0.640	0.331
**rs9951307**	**AQP4**	1.076 (0.508, 2.276)	0.849	0.783	0.684 (0.289, 1.620)	0.388	0.258	1.184 (0.529, 2.650)	0.682	0.279

All P values were calculated in relation to the major homozygote genotype. OR, odds ratios; CI, conﬁdence interval; P_adj_, adjusted p value which was corrected for those significantly associated variables in each random sampling test. Age and hypertension were adjusted in the first and fifth sampling test. Age, hypertension and LDL-C level were adjusted in the second sampling test. Age, hypertension, diabetes mellitus and LDL-C level were adjusted in the third sampling test. Age and LDL-C and cholesterol levels were adjusted in the fourth sampling test. Age, hypertension, diabetes mellitus and LDL-C and cholesterol level were adjusted in the sixth sampling test. *Signiﬁcant association with two-tailed p value <0.05. **Significant association with two-tailed p value <0.01.

## Discussion

In the present study, we only replicated the risk variant (rs699 in AGT) for LA and surprisingly found the significantly negative associations of seven polymorphisms located in *PMF1*, *ICAM1*, *TRIM65*, *FBF1* and *ACOX1* with LA in this cohort of Chinese subjects. Therefore, a strong ethnic difference is indeed implicated in the genetics of LA, and those eight variants located in six identified genes (*PMF1*, *ICAM1*, *AGT*,* TRIM65*, *FBF1*, and *ACOX1*) may be involved in Chinese white matter lesions.

As we all know, previous European GWAS and multi-ethnic GWAS revealed a lot of WMH-associated loci with genome-wide or highly suggestive significance (Fornage et al., 2011; Verhaaren et al., 2015). Among those risk loci, some variants such as rs3744028 in *TRIM65* and rs1055129 in *TRIM47* had been successfully invalidated by several candidate gene association studies in Scotland and Japan (Tabara et al., 2013; Lopez et al., 2015). Other single nucleotide polymorphisms including rs7214628 in *TRIM65* and rs78857879 in *EFEMP1* had also been verified at genome-wide significance level by one later genome-wide meta-analysis study in overall combined community populations with stroke patients of European ancestry (Traylor et al., 2016). However, most of those SNPs could not be replicated by three recently published GWAS studies in independent populations (such as whites of European descent, African Americans, or Hispanic participants) to date (Hofer et al., 2015; Traylor et al., 2016; Beecham et al., 2017). On the contrary, they identified a large number of novel WMH-associated loci including four genome-wide significant genes (*NBEAL1*, *EVL*, *C1QL1*, and *COL4A2*) and 12 suggestive genes (*CDC7*, *ST6GAL2*, *GATA6*, *GFRA4*, *TRIM6*, *WFDC3*, *FAM118A*, *UBE2C*, *PTPLA*, *RGPD4*, *ALMS1*, and *ZNF280D*), as well as four suggestive variants (rs10883817 in the introns of *AS3MT*, *CNNM2*, and* NT5C2*; rs4761974 in close proximity to *SLC4A8*; rs6135309 in the intron of *MACROD2*; and rs7664442 located in the intergenic region near *GBA3*) (Hofer et al., 2015; Traylor et al., 2016; Beecham et al., 2017).

Consistent with those genome-wide association studies and our previous replication study which aimed to verify the top risk variants identified in the first GWAS study in European population (Hofer et al., 2015; Huang et al., 2016; Traylor et al., 2016; Beecham et al., 2017), this study also did not find any significantly positive associations of those strongest and suggestive risk genes that were identified in the multi-ethnic GWAS with LA in this Chinese population. Even the strongest WMH-associated variant (rs7214628 located in *TRIM65* gene in 17q25 locus) which holds the strongest significance of *p* < 2.7E-19 and *p* < 5.1E-19 in European individuals and multi-ethnic populations, respectively (Verhaaren et al., 2015), also showed a lack of significant and detrimental effect on LA in the present study. Unexpectedly, 6 of 18 GWAS polymorphisms (rs2984613 in *PMF1*, rs2305913 in *FBF1*, rs1135640 in *ACOX1*, and rs3760128, rs7214628 and rs7222757 in *TRIM65*) were identified through the random sampling tests, and they showed significantly negative associations with LA risk. We felt surprised by this divergence among different studies and believed that this strong inconsistency could be mainly resulted from the strong ethnic differences in the genetics of LA among Europeans, Africans, Hispanics, Japanese, and Chinese. Certainly, we should not rule out other reasons, such as disequilibrium of study sample size, the effects of gender difference, age range, potential comorbid medical disorder (such as stroke), and the difference in the quantitative method and inclusion criteria of white matter lesions (or LA) among so many population-based cohorts.

Among those six GWAS polymorphisms which were identified in the present Chinese LA subjects, rs2984613 located in the intron of the read-through *PMF1-BGLAP* sequence is worth mentioning (Verhaaren et al., 2015). It was not only found to be a suggestive variant in independent European samples (*p* < 1.4E-05) (Fornage et al., 2011) but also to be a genome-wide significant locus in multi-ethnic overall samples (*p* < 2.0E-08) (Verhaaren et al., 2015). In our study, this variant also showed significant association with LA both in multiplicative model and in recessive model (*p* < 0.05). While inconsistent with the previous GWAS (Verhaaren et al., 2015), this study revealed significantly negative association of rs1984613 with LA, indicating the positive role of this variant in decreasing LA risk. Moreover, this protective effect could be validated by multiple random sampling tests. Although it did not remain significant in the tests in random cohort of 50 LA subjects but in the cohort of 220 LA subjects after adjusting for the associated variables, this protective effect of rs1984613 on LA should be suggested in the genetics of LA. Since the detecting power for this polymorphism in the cohort of 220 LA subjects is less than 80% and the small cohort size of 50 LA subjects may decrease the statistical power, it is necessary to confirm the significantly negative association of rs1984613 with LA in an independent and larger cohort. We thought that this association may show to be more significant in a larger cohort size of Chinese LA individuals, but its function should be interpreted with caution. The same as rs1984613 in *PMF1*, five other SNPs (rs2305913 in *FBF1*, rs1135640 in *ACOX1*, and rs3760128, rs7214628, and rs7222757 in *TRIM65*) showed significantly negative association with LA and represented potentially protective factors for LA. Compared with both SNPs (rs7214628 and rs7222757) showing significant association with LA only in one random sampling test, three polymorphisms (rs2305913, rs1135640, and rs3760128) were observed to be significantly associated with LA in up to three random sampling tests, and they still showed significant association in two sampling tests after controlling for the associated variables. Therefore, we preferentially recommended those variants including rs3760128 in *TRIM65*, rs2305913 in *FBF1*, and rs1135640 in *ACOX1* besides rs1984613 in *PMF1* in the genetics of Chinese LA patients followed by rs7214628 and rs7222757 in *TRIM65*.

As shown for those risk variants included in the first group, most of those 15 SNPs included in the second gene group except rs5498 in *ICAM1* and rs699 in *AGT* were not confirmed in the present Chinese cohort. However, consistent with the finding in the previous CGAS studies (Schmidt et al., 2001; van Rijn et al., 2007), rs699 showed significantly positive association with LA, indicating that AGT could act as a shared risk gene for LA between Chinese population and non-Asian population and have a detrimental effect on LA. The rs699 (M235T) polymorphism in *AGT* gene encodes threonine instead of methionine at position 235 residue of the angiotensinogen protein. It has been reported to be in complete linkage disequilibrium with one haplotype in the promoter region that could regulate the transcriptional activity in astrocytes (Paillard et al., 1999; Tsai et al., 2003; Schmidt et al., 2004). Thus, this finding in the present study supports the implication of the functional disturbance of *AGT* gene in the pathogenesis of cerebral white matter lesion. The same as rs699 in *AGT* and rs1984613 in *PMF1*, another SNP selected from previous CGAS studies, rs5498 in *ICAM1*, showed significant association with LA in up to four random sampling tests, and it still remained significant in two random tests after controlling for the associated variables. *ICAM1* gene encodes intercellular adhesion molecule 1. Its circulating level can act as one marker of endothelial dysfunction which is considered as one pathogenesis of LA (Hassan et al., 2003; Grueter and Schulz, 2012), indicating the pathogenic functions of *ICAM1* in white matter lesions. Given that the variant rs5498 (M235T) in *ICAM1* showed significantly negative association with LA in the present genetic association study, we thus think that this negative association of rs5498 with LA risk and the regulatory effect of this M235T missense on protein expression, structure, and function are required to be confirmed and explored in future.

In addition, the hypothesis that those potential variants on inflammatory genes may contribute to the increased risk of LA was also not confirmed by the findings in our present study (Lin et al., 2015). Those SNPs identified in previous studies (including rs1800795 in *IL6*, rs2290608 in *IL5RA*, rs689466 and rs20417 in *COX2*, and rs2075575 and rs9951307 in *AQP4*) (Fornage et al., 2008; Fernandez-Cadenas et al., 2011; Shan et al., 2013; Yadav et al., 2014) were not shown to be associated with LA. The inconsistent and negative results may be attributed to the following points. Firstly, the ethnic difference may contribute to the different effects of those inflammatory genes on LA. Secondly, other inflammatory factors may be involved in LA. Last but not the least, those inflammation-associated genes may not contribute to increased risk and pathogenesis of LA through the DNA variation that affects the structure and function of encoding proteins but through the abnormal expression. To demonstrate the contribution of inflammation to LA, both large scale of candidate gene association studies and genome-wide transcriptome analyses are therefore necessary to decipher those functionally inflammatory genes in the pathogenesis of LA in future.

The major strengths of our study are as follows: 1) this single SNP association analysis could avoid the noise of GWAS and improve the recalling rate of risk variants; 2) the subjects are ethnically different from those in the previous studies. However, there are still several limitations in the study. They include the following: 1) the small size of the control cohort decreases the statistical power to detect significantly associated SNPs; 2) the quadruple size difference between the control cohort and the cohort of LA subjects could leave some room for detecting false positives or false negatives; 3) except the subjects with hypertension, stroke patients included in the study cohort may interfere with the identification of potential risk genes; 4) the candidate gene association analysis has limited power to decipher so many risk genes for human complex trait (such as LA); and 5) qualitative assessment of LA is not more efficient to identify the risk SNPs than volumetric or quantitative analysis of white matter lesion. Given the lack of significant associations and those mentioned limitations in the study, we are going to perform genome-wide association analysis and transcriptome sequencing to confirm those reported risk genes and identify novel genes implicated in the pathogenesis of LA in a larger cohort of 500 LA subjects and an equivalent size of control cohort, together with scientists from other brain research centers.

## Conclusion

This is the first study to identify those risk variants for LA in a Chinese population through candidate genes-based screening approach. In this study, we did not replicate most of the risk genes that were identified from GWAS and CGAS in non-Asian populations, nor did the study confirm the putative contribution of chosen inflammatory genes to the increased risk of LA. On the contrary, this study revealed the significant associations of *PMF1*, *ICAM1*, *AGT*, *TRIM65*, *FBF1*, and *ACOX1* polymorphisms with LA and suggested strong ethnic differences in the genetics of LA. Therefore, larger scales of candidate gene screening and genome-wide sequencing study are still required to confirm and decipher the Chinese-specific risk genes for LA in China.

## Ethics Statement

This study was approved by Xiamen ethical committee, and it was registered in the Chinese clinical trial registry center (number: ChiCTR-COC-15007640). All study subjects gave informed consent.

## Author Contributions

W-QH carried out experimental design, genetic analysis, statistical analysis, and article drafting. L-LC performed genomic DNA extraction and genotyping. W-QH and H-MY revised the manuscript. QL, Q-LM, C-XL, and S-JT performed the diagnoses of LA by FLAIR-MRI and took charge of the recruitment of study subjects. W-QH and QL participated in the collection of blood samples and clinical data. QL and C-MT took responsibility in experimental design and data interpretation.

## Funding

This study was approved by Xiamen ethical committee and supported by the Science and Technology Grant of Xiamen (no. 3502Z20164002, no. 3502Z20189004), National Natural Science Foundation of China (no. 81472031, no. 81101331), and the Fujian Provincial Sanitary Bureau for the middle-aged and young backbone (no. 2015-ZQN-ZD-32; no. 2016-ZQN-81).

## Conflict of Interest Statement

The authors declare that the research was conducted in the absence of any commercial or financial relationships that could be construed as a potential conflict of interest.

## Supplementary Material

Supplemental Figure 1 Identified genotypes of 32 SNPs in 270 subjects through MALDI-TOF MS.

Supplemental Figure 2 The comparison of age and LDL-C level between subjects with LA and controls without LA.

Supplemental Table 1 Gene list for gene selection.

Supplemental Table 2 Genotyping data of 32 SNPs in 270 subjects.

Supplemental Table 3 Clinical characteristics of those randomly selected samples from the cohort of 270 subjects in six sampling tests.
