# Supplementary material for: The Associations of PMF1, ICAM1, AGT, TRIM65, FBF1, and ACOX1 Variants With Leukoaraiosis in Chinese Population
Source: Front Genet. 2019 Jul 23;10:615. doi: 10.3389/fgene.2019.00615 (PMC6664056; doi:10.3389/fgene.2019.00615)
Supplement: Supplemental Figure 1 — Identified genotypes of 32 SNPs in 270 subjects through MALDI-TOF MS. [file Datasheet_1.pdf]

The associations of *PMF1*, *ICAM1*, *AGT*, *TRIM65, FBF1* and *ACOX1* variants with leukoaraiosis in Chinese population

Wen-Qing Huang, Hui-Ming Ye, Liang-Liang Cai,
Qi-Lin Ma, Cong-Xia Lu, Sui-Jun Tong,
Chi-Meng Tzeng, Qing Lin

Corresponding addresses:

•Department of Neurology and Center for Brain Research ,
The First Affiliated Hospital of Xiamen University, No.55, Zhenhai Road, Siming District, Xiamen City, Fujian Province, China

•Translational Medicine Research Center (TMRC),
School of Pharmaceutical Sciences, Xiang'an Campus of Xiamen University, Xiang'an South Road, Xiang'an District, Xiamen City, Fujian Province, China

Supplemental Figure 1:

Identified genotypes of 32 SNPs in 270 subjects through MALDI-TOF MS.

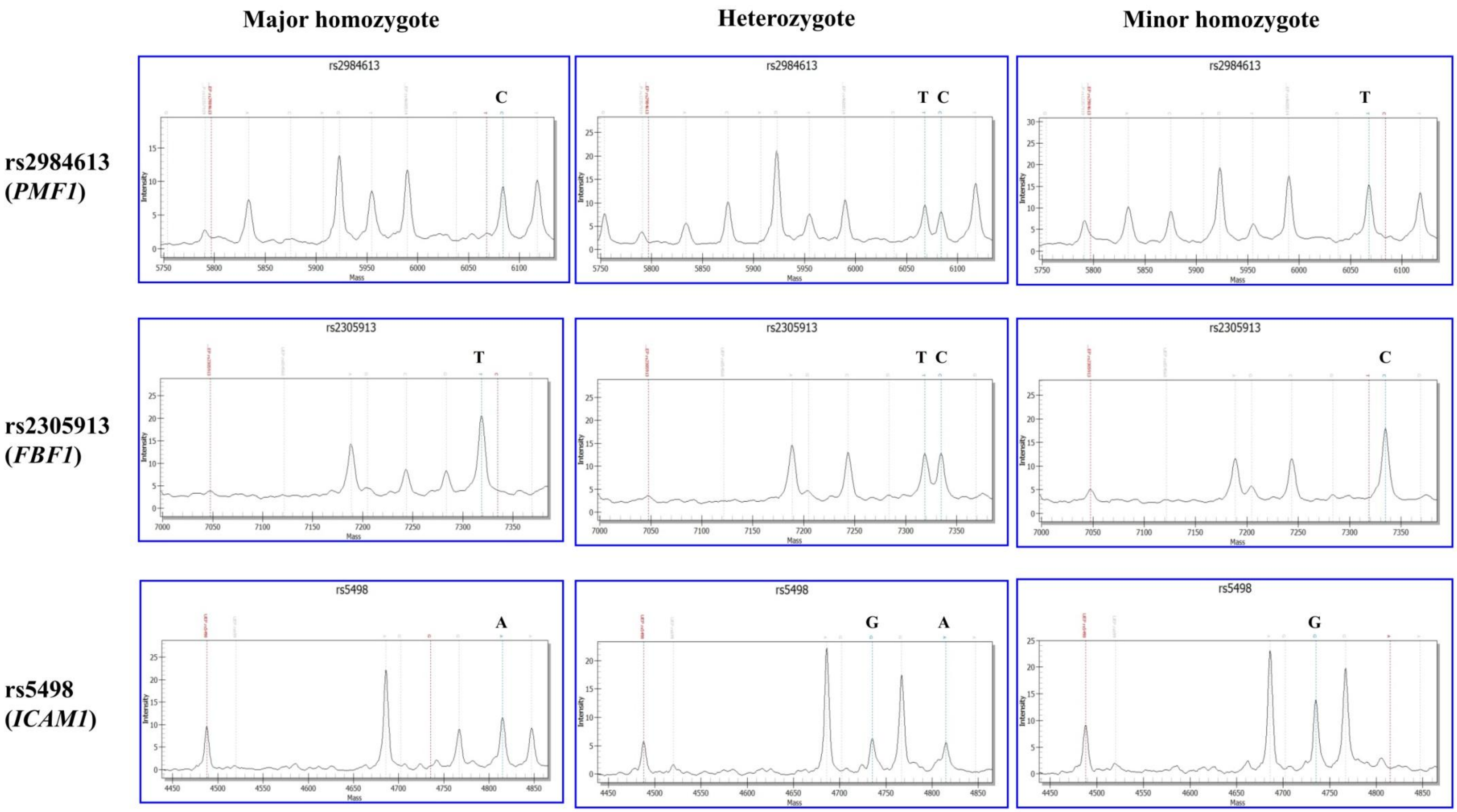

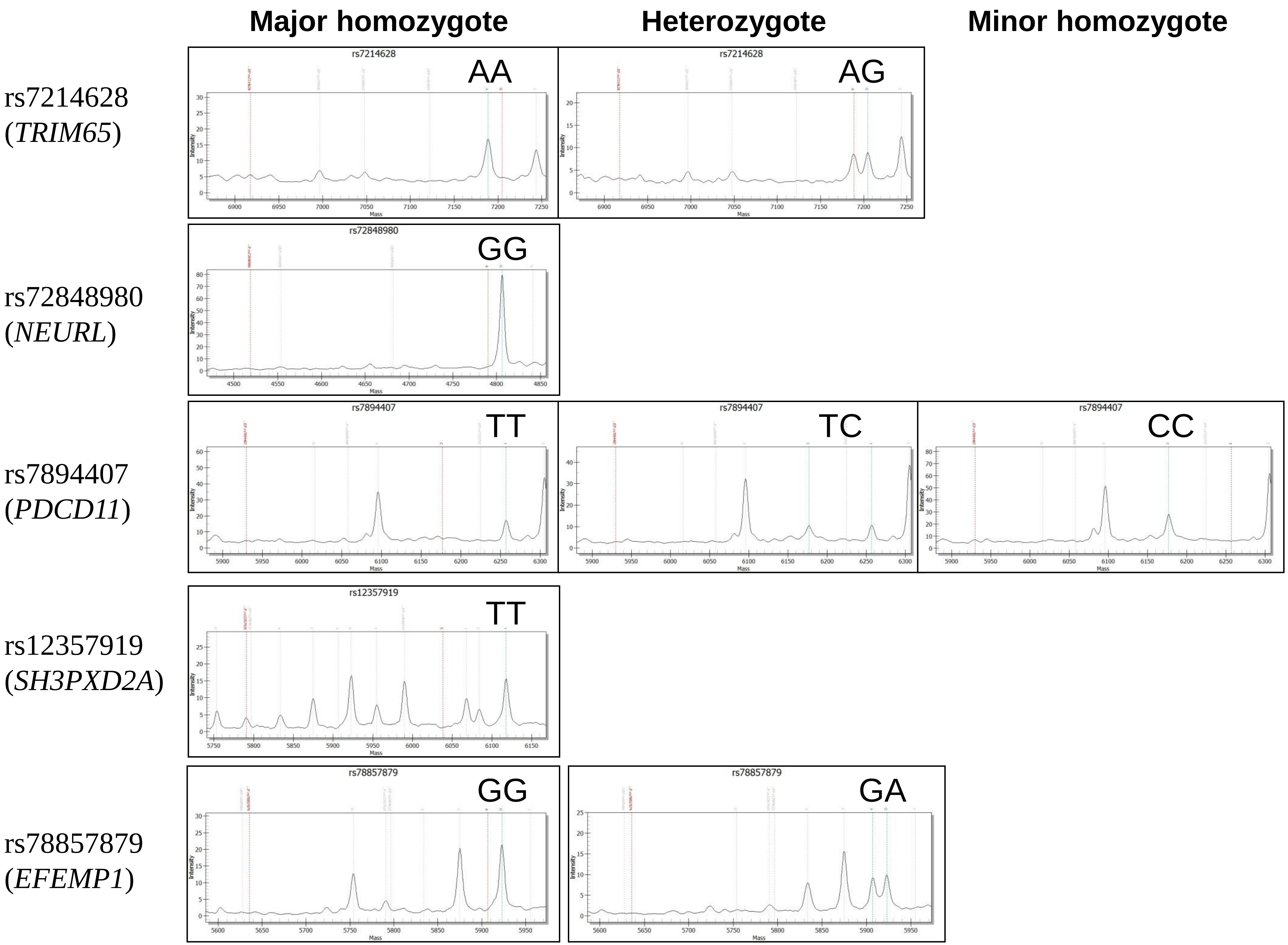

Major homozygote
Heterozygote
Minor homozygote
rs7214628 (TRIM65)
rs72848980 (NEURL)
rs7894407 (PDCD11)
rs12357919 (SH3PXD2A)
rs78857879 (EFEMP1)
rs7214628
AA
AG
rs72848980
GG
rs7894407
TT
TC
CC
rs12357919
TT
rs78857879
GG
GA
Intensity
Mass

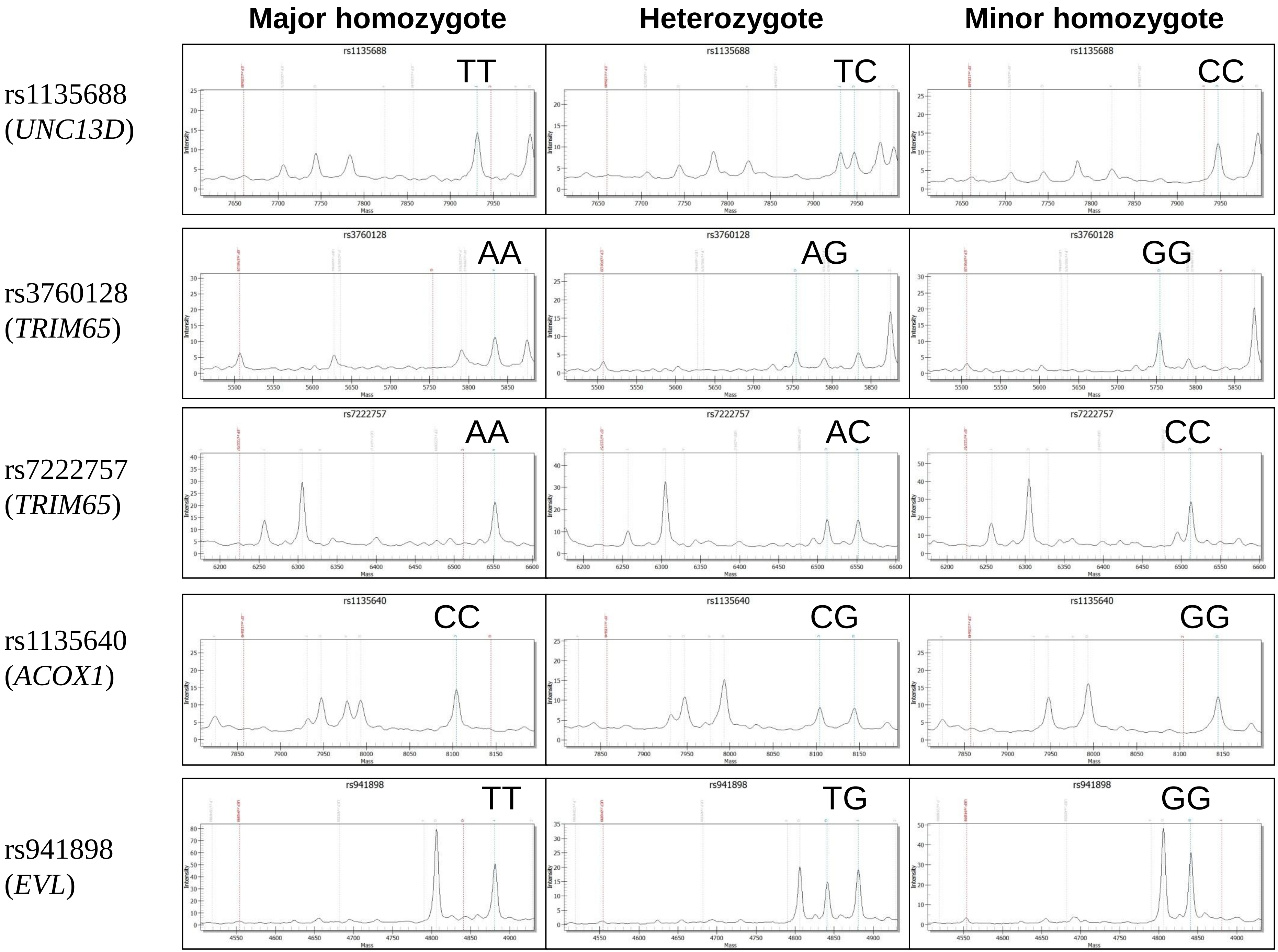

Major homozygote
Heterozygote
Minor homozygote
rs1135688 (UNC13D)
TT
TC
CC
rs3760128 (TRIM65)
AA
AG
GG
rs7222757 (TRIM65)
AA
AC
CC
rs1135640 (ACOX1)
CC
CG
GG
rs941898 (EVL)
TT
TG
GG
Intensity
Mass

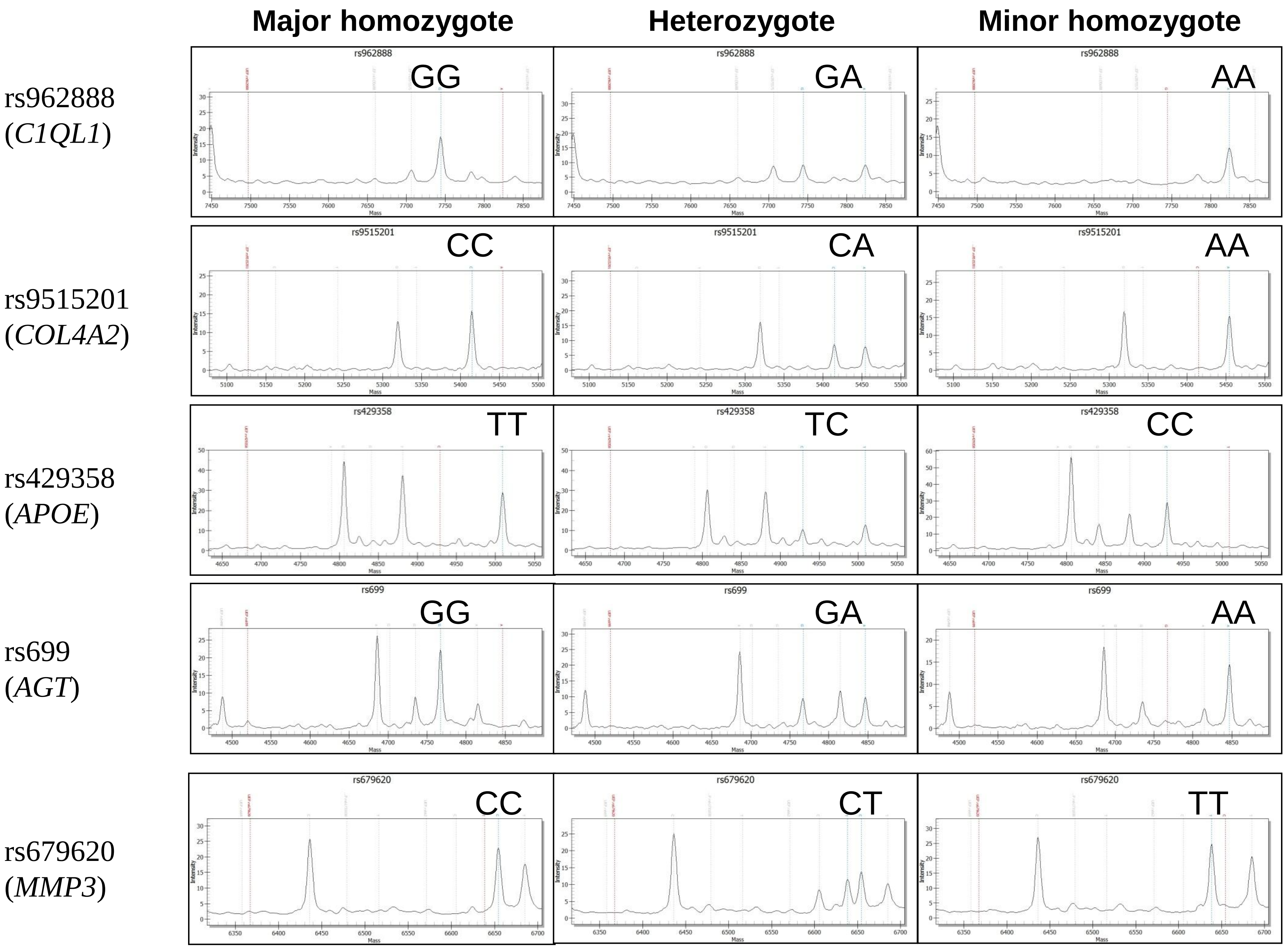

Major homozygote
Heterozygote
Minor homozygote
rs962888 (C1QL1)
GG
GA
AA
rs9515201 (COL4A2)
CC
CA
AA
rs429358 (APOE)
TT
TC
CC
rs699 (AGT)
GG
GA
AA
rs679620 (MMP3)
CC
CT
TT
Intensity
Mass

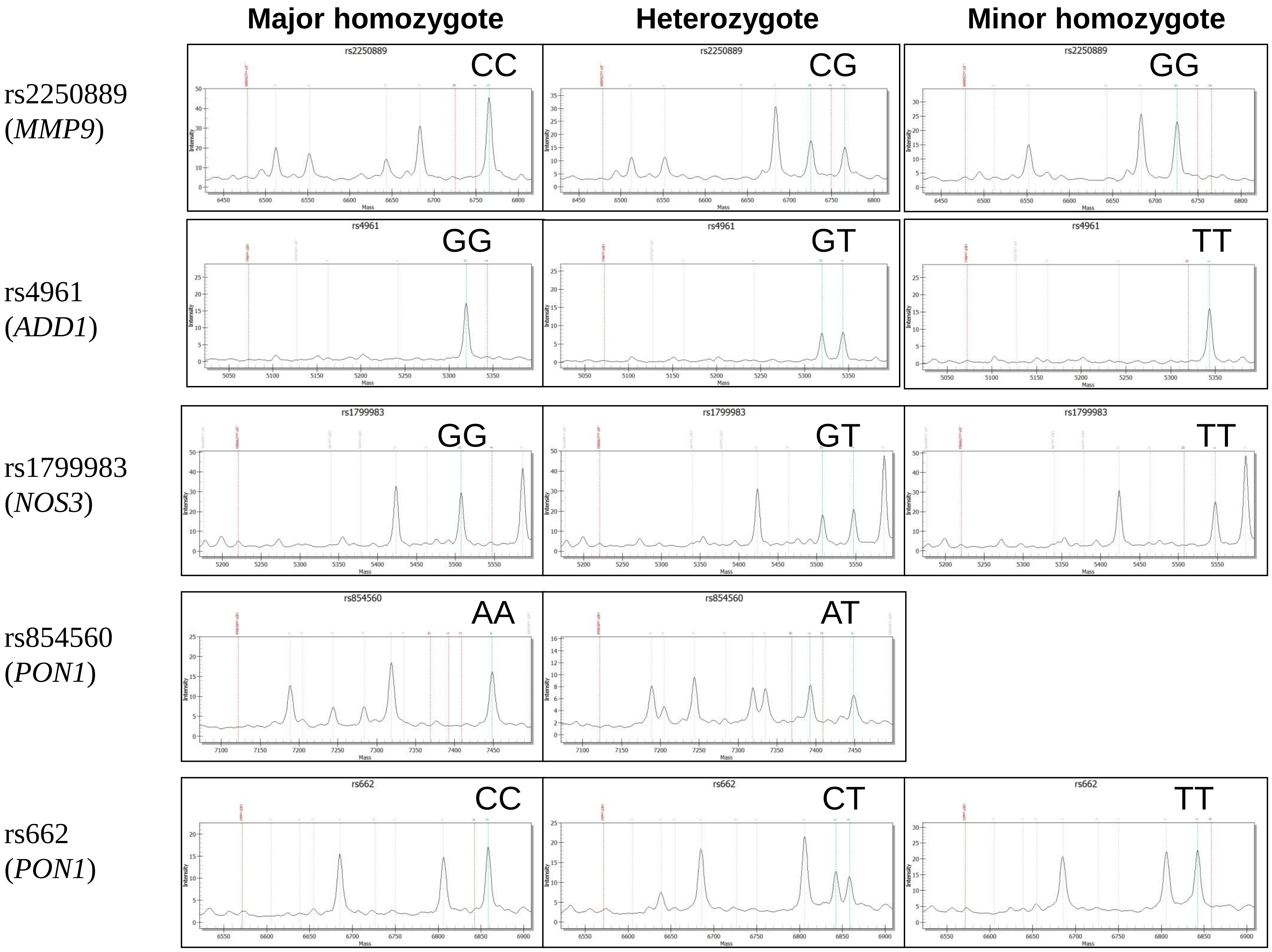
Major homozygote
Heterozygote
Minor homozygote
rs2250889 (MMP9)
CC
CG
GG
rs4961 (ADD1)
GG
GT
TT
rs1799983 (NOS3)
GG
GT
TT
rs854560 (PON1)
AA
AT
rs662 (PON1)
CC
CT
TT
Intensity
Mass

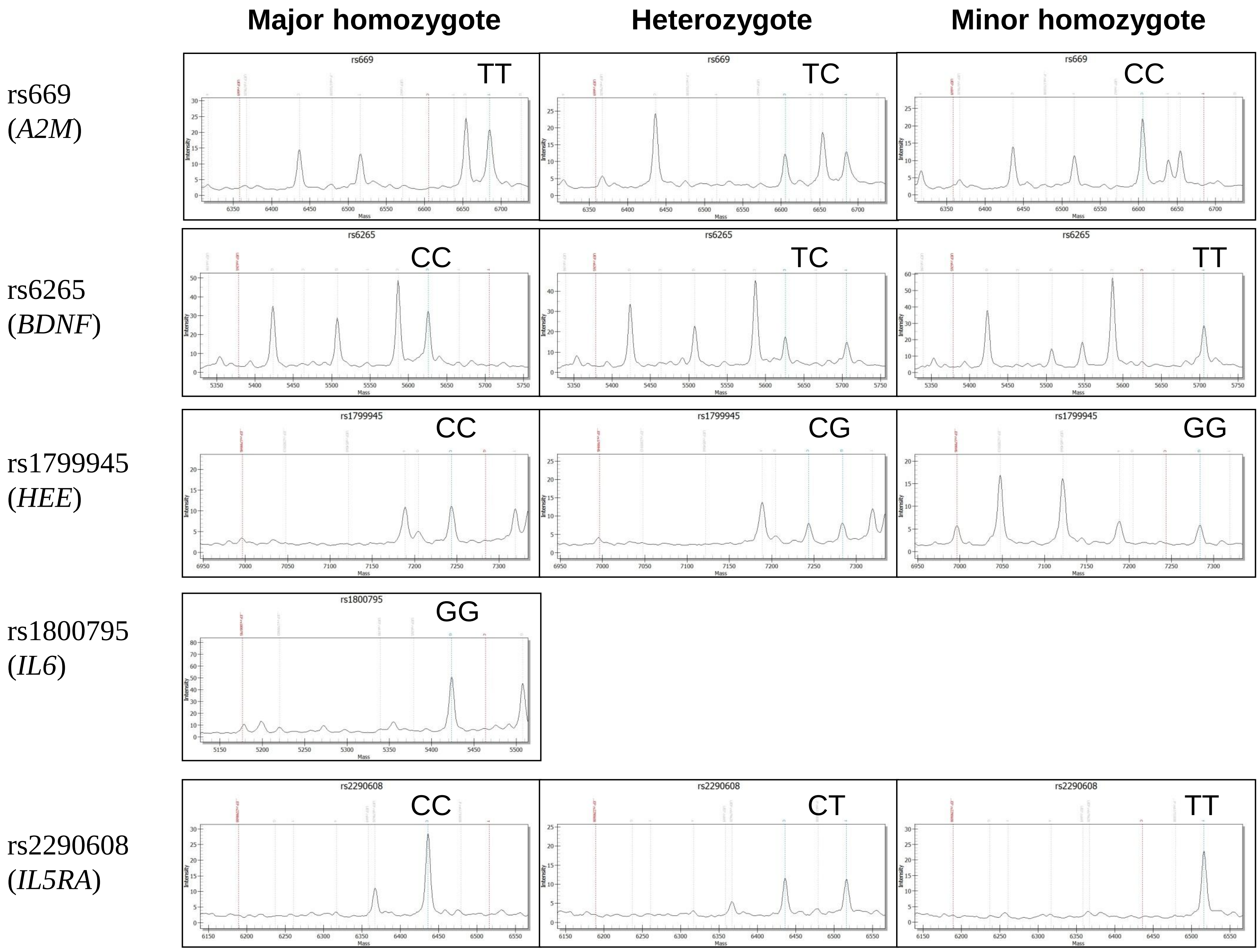
Major homozygote
Heterozygote
Minor homozygote
rs669 (*A2M*)
rs669
TT
TC
CC
rs6265 (*BDNF*)
rs6265
CC
TC
TT
rs1799945 (*HEE*)
rs1799945
CC
CG
GG
rs1800795 (*IL6*)
rs1800795
GG
rs2290608 (*IL5RA*)
rs2290608
CC
CT
TT
Intensity
Mass

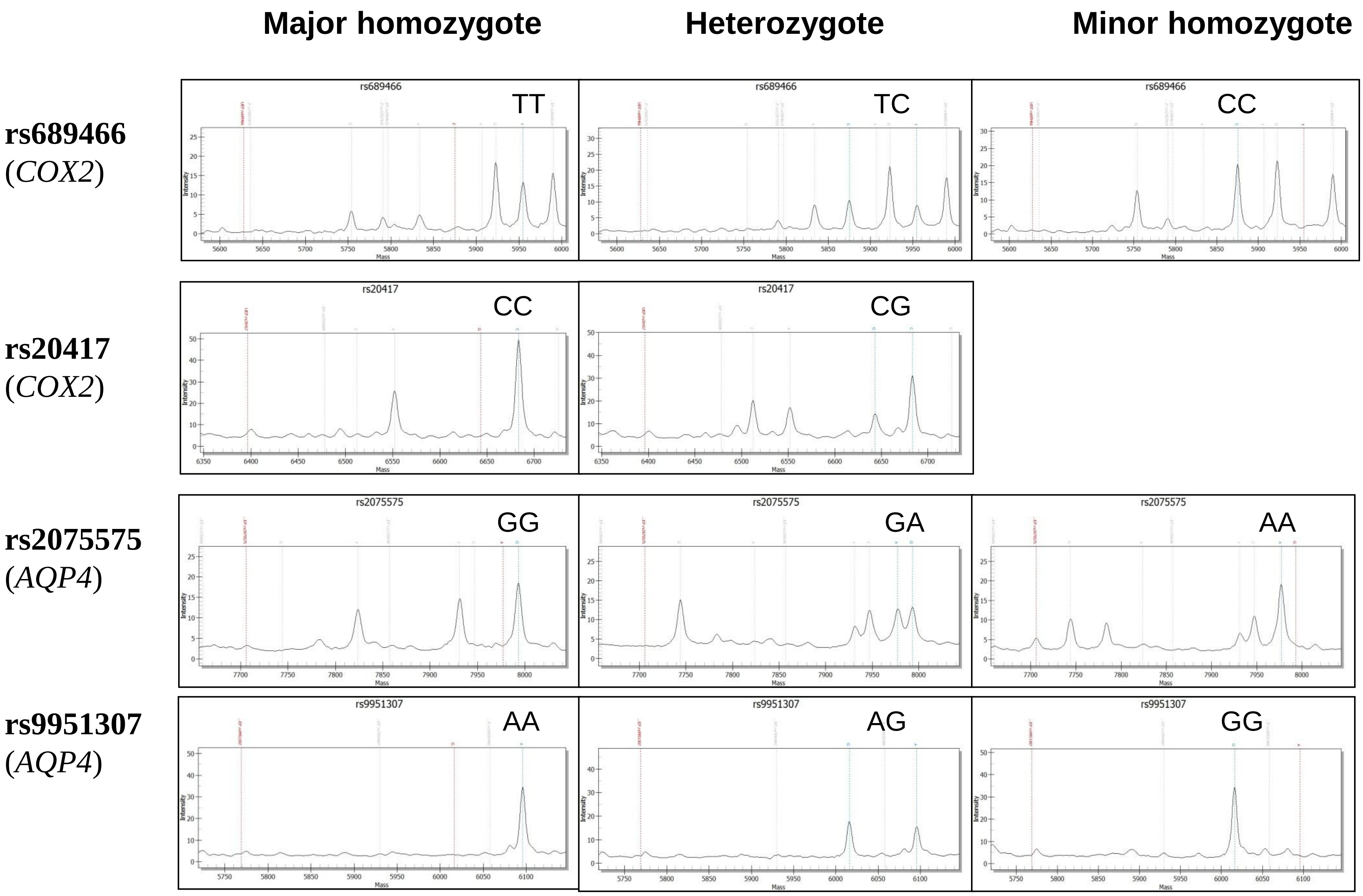
Major homozygote
Heterozygote
Minor homozygote
rs689466 (COX2)
rs20417 (COX2)
rs2075575 (AQP4)
rs9951307 (AQP4)
rs689466
TT
TC
CC
rs20417
CC
CG
rs2075575
GG
GA
AA
rs9951307
AA
AG
GG
Intensity
Mass
