# Supplementary figures and images for: The Associations of PMF1, ICAM1, AGT, TRIM65, FBF1, and ACOX1 Variants With Leukoaraiosis in Chinese Population
Source: Front Genet. 2019 Jul 23;10:615. doi: 10.3389/fgene.2019.00615 (PMC6664056; doi:10.3389/fgene.2019.00615)

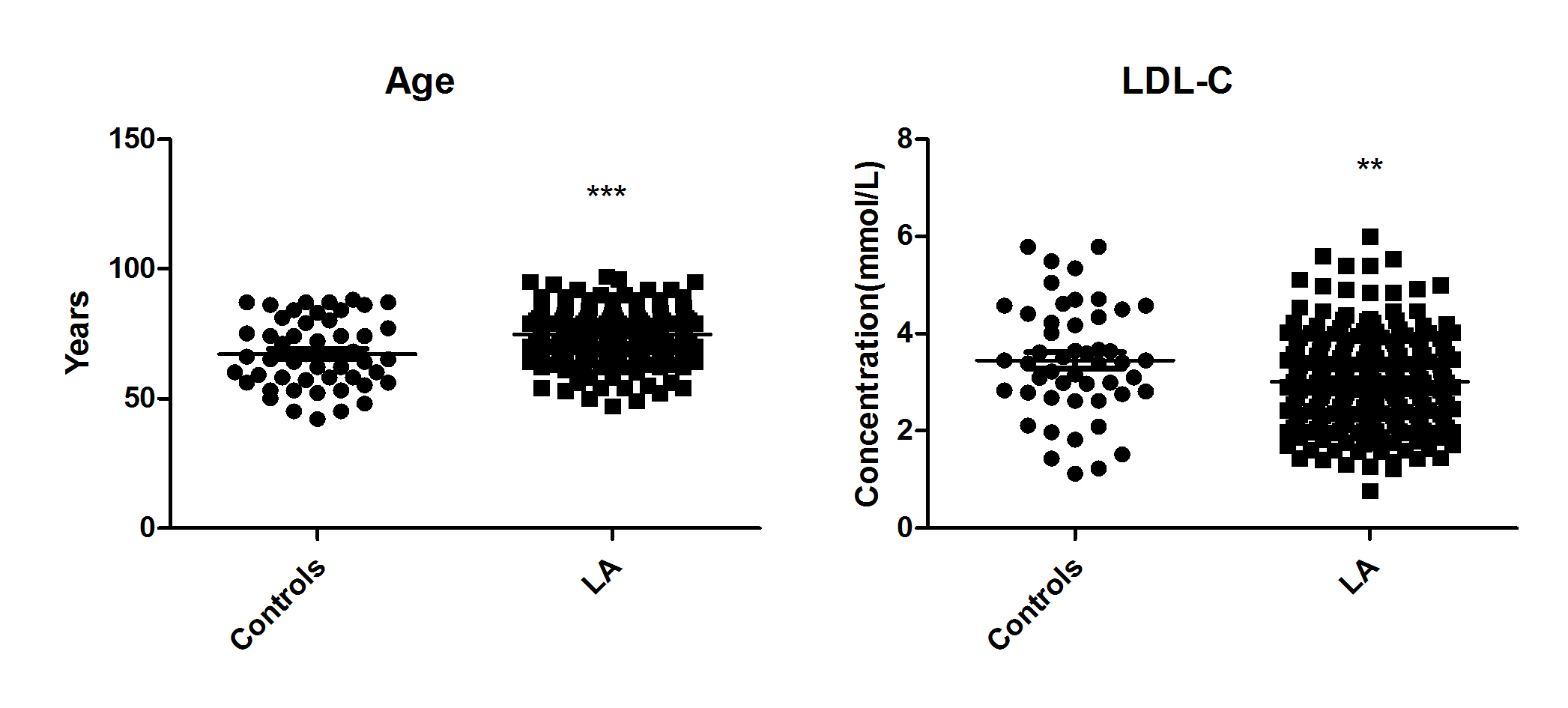

Supplement: Supplemental Figure 2 — The comparison of age and LDL-C level between subjects with LA and controls without LA. [file Image_1.jpeg]
