# Supplementary material for: The Associations of PMF1, ICAM1, AGT, TRIM65, FBF1, and ACOX1 Variants With Leukoaraiosis in Chinese Population
Source: Front Genet. 2019 Jul 23;10:615. doi: 10.3389/fgene.2019.00615 (PMC6664056; doi:10.3389/fgene.2019.00615)
Supplement: Supplemental Table 3 — Clinical characteristics of those randomly selected samples from the cohort of 270 subjects in six sampling tests. [file Table_3.doc]

| **Clinical variables** | **1st** | | | | | **2nd** | | | | |
| --- | --- | --- | --- | --- | --- | --- | --- | --- | --- | --- |
|  | **Cases (n=50)** | | **Controls (n=50)** | **OR (95% CI)** | **p-value** | **Cases (n=50)** | | **Controls (n=50)** | **OR (95% CI)** | **p-value** |
| Age (years) | 74.12±10.28 | | 67.16±13.19 | 1.051 (1.015, 1.089) | **<0.01^** a^** | 74.66±10.47 | | 67.16±13.19 | 1.054 (1.018, 1.092) | **<0.01^** a^** |
| Male, n (%) | 31 (62.0%) | | 25 (50.0%) | 0.613 (0.277, 1.358) | >0.05 | 27 (54.0%) | | 25 (50.0%) | 0.852 (0.388, 1.868) | >0.05 |
| Hypertension, n (%) | 39 (78.0%) | | 25 (50.0%) | 3.545 (1.487, 8.454) | **<0.01^**^** | 37 (74.0%) | | 25 (50.0%) | 2.846 (1.228, 6.597) | **<0.05^*^** |
| Diabetes mellitus, n (%) | 14 (28.0%) | | 7 (14.0%) | 2.389 (0.870, 6.556) | >0.05 | 12 (24.0%) | | 7 (14.0%) | 1.940 (0.693, 5.430) | >0.05 |
| HCY (µmol/L) | 16.17±6.99 | | 16.09±11.47 | 1.001 (0.959, 1.045) | >0.05 ^b^ | 16.84±7.82 | | 16.09±11.47 | 1.008 (0.967, 1.051) | >0.05 ^b^ |
| LDL-C (mmol/L) | 3.11±1.02 | | 3.45±1.16 | 0.746 (0.514, 1.083) | >0.05 ^a^ | 2.98±1.03 | | 3.45±1.16 | 0.674 (0.461, 0.985) | **<0.05^* a^** |
| Cholesterol (mmol/L) | 4.93±1.42 | | 5.22±1.42 | 0.864 (0.648, 1.154) | >0.05 ^a^ | 4.85±1.40 | | 5.22±1.42 | 0.827 (0.616, 1.111) | >0.05 ^b^ |
| Triglyceride (mmol/L) | 1.39±1.00 | | 1.34±0.83 | 1.058 (0.676, 1.656) | >0.05 ^b^ | 1.35±1.13 | | 1.34±0.83 | 1.009 (0.664, 1.532) | >0.05 ^b^ |
| HCRP (mg/L) | 6.45±4.56 | | 6.45±6.81 | 1.000 (0.931, 1.074) | >0.05 ^b^ | 7.76±4.82 | | 6.45±6.81 | 1.039 (0.965, 1.119) | >0.05 ^b^ |
| **Clinical variables** | **3^rd^** | | | | | **4th** | | | | |
|  | **Cases (n=50)** | **Controls (n=50)** | | **OR (95% CI)** | **p-value** | **Cases (n=50)** | | **Controls (n=50)** | **OR (95% CI)** | **p-value** |
| Age (years) | 74.58±9.20 | 67.16±13.19 | | 1.059 (1.020, 1.099) | **<0.01^** a^** | 74.94±8.95 | | 67.16±13.19 | 1.063 (1.023, 1.104) | **<0.01^** a^** |
| Male, n (%) | 27 (54.0%) | 25 (50.0%) | | 0.852 (0.388, 1.868) | >0.05 | 32 (64.0%) | | 25 (50.0%) | 0.563 (0.253, 1.252) | >0.05 |
| Hypertension, n (%) | 38 (76.0%) | 25 (50.0%) | | 3.167 (1.349, 7.435) | **<0.01^**^** | 28 (56.0%) | | 25 (50.0%) | 1.273 (0.579, 2.795) | >0.05 |
| Diabetes mellitus, n (%) | 17 (34.0%) | 7 (14.0%) | | 3.165 (1.176, 8.518) | **<0.05^*^** | 12 (24.0%) | | 7 (14.0%) | 1.940 (0.693, 5.430) | >0.05 |
| HCY (µmol/L) | 15.35±4.56 | 16.09±11.47 | | 0.990 (0.944, 1.039) | >0.05 ^b^ | 15.22±4.43 | | 16.09±11.47 | 0.988 (0.942, 1.036) | >0.05 ^b^ |
| LDL-C (mmol/L) | 2.89±0.94 | 3.45±1.16 | | 0.599 (0.401,0.896) | **<0.05^* a^** | 2.91±0.99 | | 3.45±1.16 | 0.624 (0.423, 0.921) | **<0.05^* a^** |
| Cholesterol (mmol/L) | 4.77±1.21 | 5.22±1.42 | | 0.769 (0.558, 1.060) | >0.05 ^b^ | 4.67±1.17 | | 5.22±1.42 | 0.720 (0.519, 0.998) | **<0.05^* c^** |
| Triglyceride (mmol/L) | 1.40±0.86 | 1.34±0.83 | | 1.094 (0.665, 1.800) | >0.05 ^b^ | 1.21±0.61 | | 1.34±0.83 | 0.765 (0.420, 1.395) | >0.05 ^b^ |
| HCRP (mg/L) | 6.41±4.62 | 6.45±6.81 | | 0.999 (0.929, 1.074) | >0.05 ^b^ | 5.06±4.31 | | 6.45±6.81 | 0.956 (0.885, 1.032) | >0.05 ^b^ |
| **Clinical variables** | **5th** | | | | | **6th** | | | | |
|  | **Cases (n=50)** | **Controls (n=50)** | | **OR (95% CI)** | **p-value** | **Cases (n=50)** | **Controls (n=50)** | | **OR (95% CI)** | **p-value** |
| Age (years) | 75.78±8.98 | 67.16±13.19 | | 1.070 (1.029, 1.112) | **<0.01^** a^** | 76.10±8.03 | 67.16±13.19 | | 1.078 (1.034, 1.123) | **<0.001^*** a^** |
| Male, n (%) | 29 (58.0%) | 25 (50.0%) | | 0.724 (0.329, 1.594) | >0.05 | 28 (56.0%) | 25 (50.0%) | | 0.786 (0.358, 1.726) | >0.05 |
| Hypertension, n (%) | 40 (80.0%) | 25 (50.0%) | | 4.000 (1.647, 9.715) | **<0.01^**^** | 37 (74.0%) | 25 (50.0%) | | 2.846 (1.228, 6.597) | **<0.05^*^** |
| Diabetes mellitus, n (%) | 15 (30.0%) | 7 (14.0%) | | 2.633 (0.967, 7.170) | >0.05 | 19 (38.0%) | 7 (14.0%) | | 3.765 (1.410, 10.051) | **<0.01^**^** |
| HCY (µmol/L) | 15.60±4.83 | 16.09±11.47 | | 0.993 (0.948, 1.041) | >0.05 ^b^ | 17.46±13.02 | 16.09±11.47 | | 1.010 (0.975, 1.045) | >0.05 ^b^ |
| LDL-C (mmol/L) | 3.14±0.90 | 3.45±1.16 | | 0.749 (0.505, 1.110) | >0.05 ^a^ | 2.96±0.89 | 3.45±1.16 | | 0.623 (0.414, 0.938) | **<0.05^* a^** |
| Cholesterol (mmol/L) | 4.88±1.12 | 5.22±1.42 | | 0.806 (0.582, 1.116) | >0.05 ^b^ | 4.72±1.15 | 5.22±1.42 | | 0.737 (0.531, 1.024) | >0.05 **^d^** |
| Triglyceride (mmol/L) | 1.35±0.60 | 1.34±0.83 | | 1.015 (0.570, 1.805) | >0.05 ^b^ | 1.22±0.58 | 1.34±0.83 | | 0.772 (0.417, 1.431) | >0.05 ^b^ |
| HCRP (mg/L) | 5.97±5.19 | 6.45±6.81 | | 0.987 (0.920, 1.058) | >0.05 ^b^ | 6.57±4.97 | 6.45±6.81 | | 1.003 (0.935, 1.077) | >0.05 ^b^ |

**Supplemental table 3. Clinical characteristics of those randomly selected samples from the cohort of 270 subjects in six sampling tests.**

**Note:** LA =leukoaraiosis, HCY =homocysteine, LDL-C =low-density lipoprotein cholesterol, HCRP: high-sensitivity C-reactive protein; OR: odds ratio, CI: conﬁdence intervals.^*^signiﬁcant association with two-sided p value<0.05, ^**^p<0.01, ^***^p<0.001. a and b indicate that the p-value estimate from binary logistic regression analysis could be confirmed by t test and nonparametric Wilcoxon test, respectively. c indicates that the p-value estimate from binary logistic regression analysis could not be confirmed by nonparametric Wilcoxon test. d indicates that the p-value estimate from binary logistic regression analysis could not be confirmed by Wilcoxon test which revealed the significant association of cholesterol level with LA instead .
